# Questiomycins, Algicidal Compounds Produced by the Marine Bacterium *Alteromonas* sp. D and their Production Cue

**DOI:** 10.3390/molecules24244522

**Published:** 2019-12-10

**Authors:** Saki Umetsu, Mamoru Kanda, Ichiro Imai, Ryuichi Sakai, Masaki J. Fujita

**Affiliations:** 1 Graduate School of Fisheries Sciences, Hokkaido University, 3-1-1 Minato-cho, Hakodate, Hokkaido 041-8611, Japan; 2 Lake Biwa Museum, 1091 Oroshimo-cho, Kusatsu, Shiga 525-0001, Japan

**Keywords:** algicidal bacteria, questiomycins, phenoxazinone antibiotics, *Alteromonas*, *Chattonella antiqua*, harmful algal bloom, red tide

## Abstract

Questiomycin A (**1**) along with three new compounds, questiomycins C–E (**2**–**4**), were isolated from culture of *Alteromonas* sp. D, an algicidal marine bacterium, guided by algal lethality assay using the raphidophyte, *Chattonella antiqua*, one of the causative organisms of harmful algal bloom. The structures of **1**–**4** were assigned on the basis of their spectrometric and spectroscopic data. Compounds **1** to **4** exhibited algicidal activity against *C. antiqua* with LC_50_ values ranging from 0.18 to 6.37 μM. Co-cultivation experiment revealed that **1** was produced only when the microalgae and the bacterium are in close contact, suggesting that some interactions between them trigger the biosynthesis of questiomycins. These results suggested that the algicidal bacteria such as *Alteromonas* sp. D can control microalgae chemically in marine ecosystem.

## 1. Introduction

Red tide, also known as harmful algal bloom (HAB), causes tremendous economic damage on the fisheries industries, and threatens coastal environment even human health [1,2,3,4,5,6]. Massive proliferation of certain algal species, such as the raphidophytes *Chattonella antiqua*, *Heterosigma akashiwo*, the dinoflagellates *Karenia mikimotoi*, *Alexandrium tamarense*, and the diatoms *Eucampia zodiacus*, *Pseudo-nitzschia australis*, are responsible for HAB [7]. Local eutrophication of coastal water, accompanied by economic development, is likely major factor to boost HABs, however recent climate change may enhances the risk of HAB globally [2,3]. To date, chemical and physical countermeasures such as cray dispersal and ultrasonic irradiation [8,9,10] have been used in attempts to control HAB. Although these methods are useful in certain areas [8], problems regarding effectiveness, cost, and secondary pollution are yet to be solved, and development of an alternative strategy is demanded.

Recently, marine bacteria that potently lyse microalgae, called algicidal bacteria, draw a great deal of attention as an environmentally friendly strategy to mitigate and prevent HAB [11]. Numbers of algicidal bacteria have been separated from various marine sources, and their taxonomy, distribution, and target microalgae are extensively studied [10,12,13,14]. Most of algicidal bacteria reported to date belong to phyla Proteobacteria and Bacteroidetes, which are common taxa in the marine environment suggesting that algicidal bacteria are one of the key players in coastal ecosystems [12,13,14]. For example, algicidal bacteria are found in high density in the biofilm on seagrasses, suggesting that the algicidal bacteria inhabit the seagrass-borne biofilm, and are supplied to the coastal environment [6,15,16,17]. Algicidal bacteria attack algal cells in either direct (require attachment on the target algal cells) or indirect (release active molecules) manners [13,14]. The former uses some hydrolases, such as proteases and glycosidases, to lyse a variety of microalgal species [18,19,20,21]. Several low-molecular-weight compounds have also been identified as algicidal principals [12,13,22,23,24]. These molecules are released from the bacteria into the water, and these dispersed algicidal agents attack algal cells. Small molecular algicidal compounds tend to show some target specific activity. These findings encouraged the development of biological strategies to mitigate HABs, however the mechanisms and chemical basis of many algicidal bacteria are still left to be investigated. In the present study, we identified four aminophenoxazinone alkaloids, questiomycins A and C to E from *Alteromonas* sp. D, one of the first reported algicidal bacteria [11]. Here, isolation, structure elucidation, and algicidal activities of questiomycins are described. We also propose plausible chemo-ecological basis of algicidal actions of questiomycins triggered by close contact between the alga and bacterium.

## 2. Results and Discussion

### 2.1. Purificatin and Structure Elucidation

**Figure 1:**
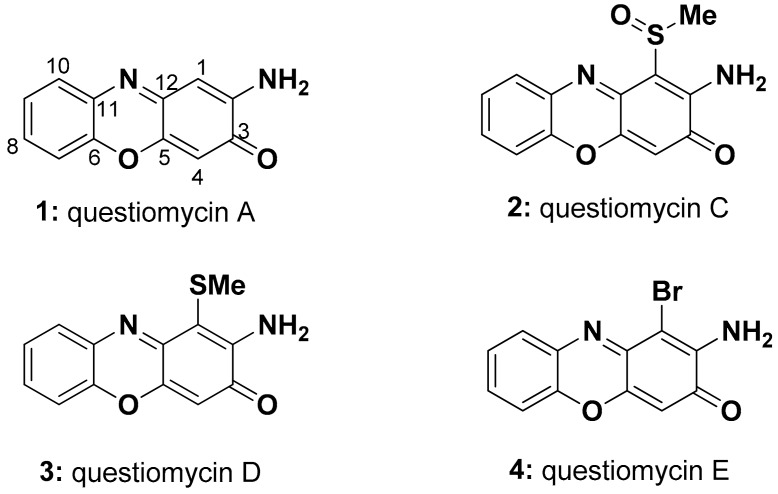
Aminophenoxazinone-based algicidal molecules, questiomycin A (**1**) and new analogues C–E (**2**–**4**), from the marine bacterium *Alteromonas* sp. D.

A total 1.6 L of *Alteromonas* sp. D culture was grossly separated by a reversed-phase C18 vacuum liquid chromatography. The active molecules eluting between 70% and 100% MeOH fractions were combined and further chromatographed on a Sephadex LH-20 column. The orange-colored band was collected and purified by reversed-phase HPLC to afford questiomycin A (2-aminophenoxazin-3-one, APO, (**1,** Figure 1)) as a major active compound [25]. Spectral data for **1** agreed well with those reported previously [26] (Table 1 and Table 2, Supplementary Materials Table S1). Three new compounds, questiomycins C–E (**2**–**4**, Figure 1), which exhibited nearly identical UV spectra and color with those of **1**, were also purified as minor analogues from an additional large scale culture.

**Figure 2:**
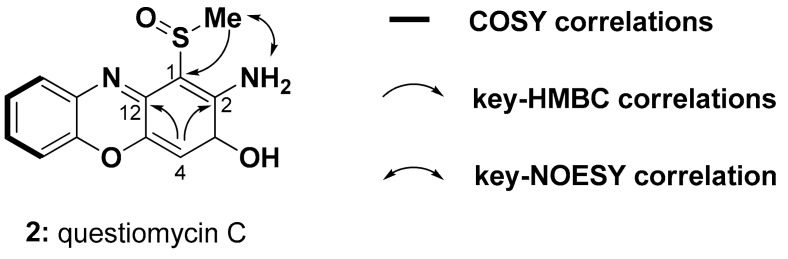
Two-dimensional NMR data assignment of questiomycin C (**2**).

Molecular formula of questiomycin C (**2**) was deduced as C_13_H_10_N_2_O_3_S on the basis of its HR-ESI-MS data, which was CH_2_OS larger than that of compound **1**. An NMR spectral profile of **2** was quite similar to that of **1** (Table 1 and Table 2), except one of the two singlet aromatic proton signals corresponding to H-1 or H-4 in **1** was missing in **2**. Instead, a methyl singlet was observed at *δ*_H_ 2.98 suggesting that either C-1 or C-4 was substituted by a methyl sulfoxide group. Key HMBC correlations from *δ*_H_ 6.46 (H-4) to C-2 and C-12 indicated that the H-1 in **1** was replaced by the methyl sulfoxide group in **2** (Figure 2). NOESY cross peaks between methyl sulfoxide and NH_2_ protons (Supplementary Materials Figure S11) assigned the structure of questionamycin C (**2**) as 2-amino-1-methylsulfoxy-phenoxazin-3-one. An absence of optical rotation suggested that **2** is likely racemic at the chiral center of the sulfoxide group.

Molecular formula of questiomycin D (**3**) was determined as C_13_H_10_N_2_O_2_S from the HR-MS data, which was one oxygen smaller than **2**. The ^1^H and ^13^C NMR spectra was almost identical with those of **2** (Table 1 and Table 2), but methyl signal was shifted from *δ*_H_ 2.98/*δ*_C_ 39.0 to *δ*_H_ 2.31/*δ*_C_ 17.1. Considering the difference of the chemical shifts as well as molecular formula, it was strongly suggested that methyl sulfoxide group in **2** was replaced by a methyl sulfide group in **3**. Detailed analyses of 2D NMR spectra including HMBC and NOESY data revealed the substitution position to be C-1 as **2** (Supplementary Materials Figures S16 and S17). Thus, the structure of questiomycin D (**3**) was elucidated as 2-amino-1-methylthio-phenoxazin-3-one.

An ESI-MS spectrum of questiomycin E (**4**) showed molecular ion peaks at *m/z* 290.95 and 292.95 with nearly same ratio indicating the presence of a bromine atom. The molecular formula for **4** was revealed as C_12_H_7_N_2_O_2_Br on the basis of HR-ESI-MS data. All NMR spectra including 2D data were very similar to those of **2** and **3** except for an absence of a methyl signal, and the high field shift of C-1 due to the shielding effect of bromine atom. Based on the spectral data above, it was concluded that structure of questiomycin E (**4**) is 2-amino-1-bromo-phenoxazin-3-one as shown in Figure 1.

Questiomycin A (**1**) has been reported from various bacterial species including the terrestrial Actinomycete [25], the marine bacteria *Halomonas* sp. [27], and *Actinomadura* sp. [26] as a cytotoxic and antibiotic compound. Additionally, compound **1** is known as an allelochemical agent of the terrestrial plants belonging to the family Poaceae, including wheat and corn, to suppress the growth of the competing weeds [28,29]. A 2-aminophenoxazin-3-one (APO) core structure of questiomycins is also found in many bioactive secondary metabolites including grixazone [30], chandrananimycins [26], and actinomycins [31], a clinically used anti-cancer agent.

It is reported that 2-aminophenoxazin-3-one (APO) core structure of bacterial products can be formed by oxidative coupling of two *o*-aminophenols catalyzed by various types of oxidases [32,33,34]. We therefore supplemented *o*-aminophenol to the *Alteromonas* sp. D culture medium to obtain larger amount of questiomycins. As expected, the supplemented culture provided compounds **1** and **4** with the concentration of 7 and 158 times as high as that in the normal conditions, respectively. Compounds **2** and **3**, however, were not increased even after supplementation of sulfur sources such as dimethylsulfide and dimethyldisulfide in *o*-aminophenol enriched medium (Supplementary Materials Figures S23 and S24).

### 2.2. Biological Activities 

Algicidal activity of compounds **1** to **4** was evaluated using *C. antiqua* (Figure S25). Compounds **1** to **4** showed algicidal activity in concentration-dependent manner, with LC_50_ values of 0.64, 0.18, 6.37, and 0.20 μM, respectively (Table 3, Figure S26). Although compounds **1**, **2**, and **4** were equally active, questiomycin D (**3**), with methylthioether at positin-1 was significantly less active than three other congeners. General toxicity of **1** was evaluated using the dinoflagellate *Karenia mikimotoi*, the diatom *Chaetoceros didymus*, the edible red macroalga *Bangia fuscopurpurea*, the brine shrimp *Artemia salina*, and the freshwater Medaka fish *Oryzias* sp. Toxicities of **4** to the brine shrimp were also tested. Compound **1** was toxic to all the organisms tested, especially to microalgae, but it was less toxic to multicellular agla and aquatic animals (Table 3, Figures S27 and S28). 

Although **4** was highly toxic to *C. antiqua*, it did not kill the brine shrimp even at the highest concentration tested (68.7 μM), which is nearly saturated concentration (Table 3, Figure S28). These results showed that the questiomycins have potent lethal activity against microalgae but are less toxic against other aquatic organisms. Interestingly, substitution of C-1 position with bromine atom did not affect the activity against *C. antiqua*, but toxicity to a crustacean was largely decreased. 

### 2.3. Co-Cultivation of C. antiqua and Alteromonas sp. D

The results from the above experiments strongly suggested that the algicidal action of *Alteromonas* sp. D is ascribable, at least in part, to compounds **1** to **4**; however, these compounds were produced in cultivation conditions completely different from those of the actual marine environment. We thus tested the production of questiomycins and their algicidal actions in the condition similar to those in microalgae blooming environment. *C. antiqua* was co-cultiured with *Alteromonas* sp. D in the modified SWM-3 medium where no excessive nutrients were contained. Two days after inoculation, most algal cells died and precipitated on the bottom of the flasks, while the *C. antiqua* culture without strain D showed normal growth (Figure S29).

**Figure 3:**
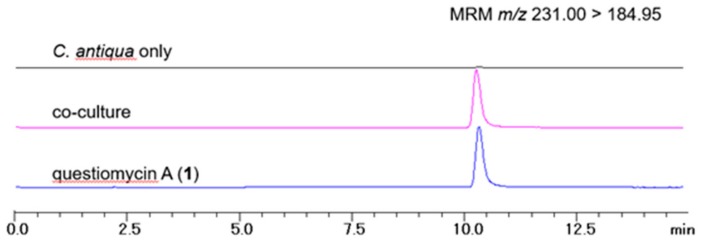
LC-MS chromatograms of the extracts of *C. antiqua* culture without strain D (black), co-cultivation (pink), and questiomycin A (**1**) standard (blue). Calculated concentration of compound **1** in co-cultivation medium was 0.31 ng/mL.

LC-MS analyses of the extracts of the above co-cultivation media and algal cells revealed that compound **1** was indeed produced in the condition similar to the algal blooming sea water (Figure 3). However, its calculated concentration was 0.31 ng/mL (1.46 nM), which is about 400 times lower than LC_50_ value of compound **1**. Note that other questiomycins **2**–**4** were not detected in this experiment. Although the activity of **1** in two separate set of experiments are not comparable directly, due to differences in initial algal cell density as well as cultivation period, the large difference in the concentration suggested to us some mechanistic insight into the action of **1**. 

**Figure 4:**
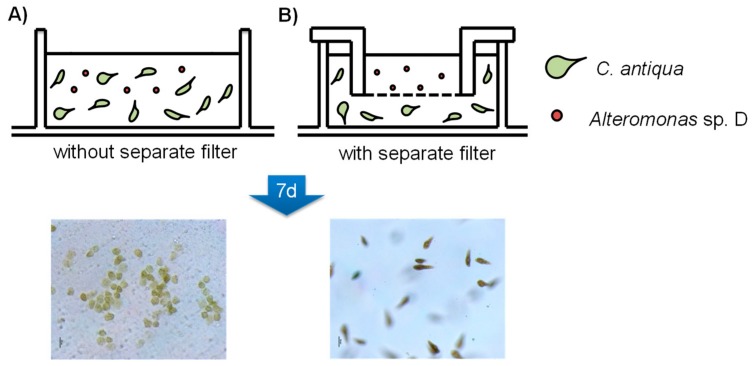
Co-cultivation of *Alteromonas* sp. D and *C. antiqua* without separate filter (**A**) and co-cultivation with separate filter (**B**). Pore size 0.4 μm membrane was used.

We therefore hypothesized that the significant difference between the LC_50_ value and detected concentration of **1** in the above experiment is due to production of algicidal materials other than questiomycics by *Alteromonas* sp. D, or the strain D produced **1** upon close proximity to the microalgae, and topical action of **1** lead to lyse the algal cells [35,36]. In the latter case, *Alteromonas* sp. D can kill *C. antiqua* only in contact with the algal cells, but not under the condition where the bacterium was separated from the alga by semipermeable membrane. To test this idea, *C. antiqua* and strain D was co-cultivated with or without separation membrane (Figure 4), in that the bacterium and algae can freely contact physically and chemically in condition-A, but in condition-B, only chemical contact was possible between the organisms. After seven days from bacterium inoculation, all *C. antiqua* cells lysed in the condition-A wells, however cells in the condition-B survived (Figure 4). LC-MS analyses showed that concentration of **1** in the condition-A media was 1.5 nM, which was nearly identical to the previous co-cultivation experiment, while that in the condition-B medium was less than detection limit, indicating that no or trace amount of questiomycins were produced in the separated culture (Figure S30). 

These results could not rule out the possibility of presence of the alternative algicidal molecules, but strongly supported an idea that physical contact between microalga and algicidal bacterium would be one of the triggers to produce algicidal molecules such as questiomycins under the oligotrophic environment, and thus only a small amount of algicidal compound is enough to kill the cells in contact. This mechanism is apparently cost efficient for the algicidal bacterium, and also beneficial for using the strain for preventing HAB due to the fact that the least amount of toxic molecules would spread in the environment. Mechanisms of algicidal activity by questiomycins as well as biosynthetic regulation of algicidal molecules in the bacterium are intriguing questions to be answered. Furthermore, the presence and function of questiomycins in actual environment need to be investigated. Nevertheless, our findings, identification of the algicidal molecules in the algicidal bacteria, would be of great help to further understand the chemical-ecological phenomena occurring in HAB.

## 3. Materials and Methods

### 3.1. General Experimental Procedures

The 1D and 2D NMR data including ^1^H, ^13^C, COSY, HSQC, HMBC, and NOESY spectra were recorded on a JNM-ECZ400S/L1 NMR spectrometer (JEOL, Tokyo, Japan) at 400 MHz for ^1^H and 100 MHz for ^13^C using the default pulse-sequence parameters preset in the spectrometer. Chemical shifts of ^1^H NMR spectra were referenced to the solvent peaks: *δ*_H_ 2.49 and *δ*_C_ 39.5 for DMSO-*d*_6_. Preparative and analytical HPLC were performed with a Prominence HPLC system equipped with photodiode array detector (Shimadzu, Kyoto, Japan). LC-MS analyses were carried out with an LCMS-8040 LC-MS system (Shimadzu). High-resolution electrospray mass spectra (HRESIMS) were obtained on a Sciex Triple TOF^+^5600 mass spectrometer (AB SCIEX, Framingham, MA, USA). UV spectra were measured on a SpectraMax M2 UV spectrophotometer (Molecular Devices, Sunnyvale, California, USA). Cell density of *C. antiqua* were measured by 10-AU Fluorometer (Turner Design, San Jose, California, USA). All chemicals were purchased from FUJIFILM Wako Pure Chemical (Osaka, Japan), Nacalai Tesque (Kyoto, Japan), or Takara Bio (Shiga, Japan), except for those specifically mentioned.

### 3.2. Biological Materials

*Alteromonas* sp. D (16S rRNA sequence: accession number AB040466) was isolated from sea water of Hiroshima-bay (Hiroshima, Japan) in 1991 [11]. The strain was deposited as glycerol stock until use. An axenic strain of the raphidophycean flagellate *C. antiqua* (NIES-1, provided from the National Institute for Environmental Study) was used. Algal strain was maintained in the modified SWM-3 medium (Table S2) at 20 °C under a light intensity of 50–100 μmol photons m^−2^s^−1^ using a 14:10 h light–dark photocycle. Axenic culture of the dinoflagellate *K. mikimotoi* and the diatom *C. didymus* were maintained in the modified SWM-3 medium, and incubated under 14 h light, 10 h dark photocycle condition at 22 °C. Filamentous red-macroalgae, *B. fuscopurpurea*, was maintained in artificial sea water at 20 °C. Cysts of the brine shrimp, *A. salina*, were purchased from Japan Pet Design (Tokyo, Japan) and kept at 4 °C until use. The Medaka fish, *Oryzias sp.*, were purchased from a market in Hakodate city and kept at 20 °C.

### 3.3. Cultivation of Bacterium, Extraction, and Isolation of Algicidal Compounds

*Alteromonas* sp. D was pre-cultured in 10 mL ST medium (trypton 5 g/L, yeast extract 0.5 g/L in artificial sea water), inoculated into ST medium (200 mL in 1 L Erlenmeyer flasks, 8 flasks in total), and was shaken (215 rpm) for four days at 25 °C. The conditioned medium was centrifuged, and the supernatant was passed through C18 reversed-phase column (5 × 20 cm), eluting with 0%, 20%, 50%, and 70% aqueous MeOH (500 mL each), then by 100% MeOH (500 mL). Fractions eluted with 70 and 100% MeOH were combined and concentrated, followed by fractionated by a gel filtration column using Sephadex LH-20 (4 × 100 cm, GE Helthcare, Chicago, Illinois, USA) using MeOH as an eluent. An active fraction obtained as an orange-colored band was further purified by reversed-phase HPLC (Develsil C30-UG-5, Nomura Chemical, Aichi, Japan; gradient elution of 32–100% MeOH) to afford compound **1** (1.25 mg) as a major active compound. From a large-scale (4 L) culture, three new compounds, **2** (1.51 mg), **3** (1.03 mg), and **4** (0.79 mg) along with **1** (13.1 mg) were isolated.

Questiomycin C (**2**): Orange powder; UV (DMSO) λ_max_ 230 nm (ε 5.00 × 10^4^), 428 nm (ε 1.85 × 10^4^); NMR data, see Table 1 and Table 2; HR-ESIMS m/z 275.0482 (M + H)^+^ (calcd. for C_13_H_11_N_2_O_3_S, –0.8 mmu).

Questiomycin D (**3**): Orange powder; UV (DMSO) λ_max_ 228 nm (ε 6.76 × 10^4^), 428 nm (ε 1.97 × 10^4^); NMR data, see Table 1 and Table 2; HR-ESIMS m/z 259.0537 (M + H)^+^ (calcd. for C_13_H_11_N_2_O_2_S, –0.4 mmu).

Questiomycin E (**4**): Orange powder; UV (DMSO) λ_max_ 241 nm (ε 2.54 x 10^4^), 426 nm (ε 2.54 × 10^4^); NMR data, see Table 1 and Table 2; HR-ESIMS m/z 290.9757 (M + H)^+^ (calcd. for C_12_H_8_N_2_O_2_Br, –1.2 mmu).

### 3.4. Preparation of Questiomycin A (***1***) and E (***4***) by Supplemented Fermentation with o-Aminophenol

Filter sterilized ethanol solution of *o*-aminophenol was added (final concentration 100 mg/L) into *Alteromonas* sp. D overnight culture (0.2 L of 1/2-ST medium; trypton 2.5 g/L, yeast extract 0.25 g/L in artificial sea water), then further shaken for four days at 28 °C (180 rpm). Cultured broth was subjected to the repetitive fractionation including C18 column chromatography, Sephadex LH20 size exclusion chromatographym, and silica gel column chromatofgraphy to obtain compound **1** (4.5 mg) and **4** (6.2 mg). These materials were used for biological assays.

### 3.5. Algicidal Assay Using C. antiqua

The axenic cultures of C. antiqua was diluted with modified SWM-3 medium (Table S2) to a cell density of 1–3 × 10^3^ cells mL^−1^, and 0.8 mL aliquots were transferred into each well of sterilized 48-well microtiter plates, and kept for 1 day. Then, 8 μL of test samples (1 mg/mL in DMSO) were added to the wells, and then incubated for 1 week. Pure DMSO was used as a negative control. Each well was observed daily with an inverted microscope (Nikon ECLIPSE TE200, Tokyo, Japan). Algicidal activity was evaluated based on the change of the cell morphology and motility and used for activity guided separation.

For quantification assay, 4 μL of test samples (1.000, 0.333, 0.111, 0.370, 0.123, 0.041 μg/mL in DMSO) were added to the test tubes containing *C. antiqua* culture (4 mL of 3 *×* 10^3^ cells/mL), and then the tubes were incubated for seven days under the same condition above. Pure DMSO (4 μL) was used as a control. Cell density was monitored by measuring autofluorescence of live motile cells using 10AU Fluorometer. Medium without alga was used as background. All experiments were performed in triplicate. The LC_50_ and 95% confidence interval values were calculated using GraphPadPrism8 (GraphPad Software, San Diego, CA, USA) with non-linear four parameter logistic regression (vs. log concentration) to the sigmoid curve by non-linear least squares.

### 3.6. Algicidal Assay against Karenia mikimotoi and Chaetoceros didymus

Axenic culture of the dinoflagellate *Karenia mikimotoi* and the diatom *Chaetoceros didymus* were diluted with a modified SWM-3 medium (1–3 × 10^3^ cells mL^−1^), and 792 μL aliquots were transferred into wells of 48-well microtiter plates and incubated at 22 °C overnight. Two-fold serial dilution of the test compound (8 μL of 1.000, 0.500, 0.250, 0.125, 0.063, 0.031,0.016, 0.008 mg/mL in DMSO) was added to each well, and then incubated further for seven days. Microscopic observation was carried out using the inverted microscope to observe cell mortility and morphology. Algal cells, which lost mortility and had observable deformation including cytosol shrinking or leakage, loss of chrolophyl, burst of the thecal plates, or frustules, were counted as dead cells (Figure S27). All tests were done in triplicate. 

### 3.7. Artemia salina Assay

*Artemia salina* (brine shrimp) eggs were inoculated in sea water with aeration at room temperature for 24 hours. Hatched larvae were transferred into 24-well plates containing 792 μL sea water (10 larvae/well), then 8 μL of test samples (compounds **1** and **4**; 2.000, 1.000, 0.500, 0.250, 0.125, 0.625 mg/mL in DMSO) were added. Pure DMSO (8 μL) was used as a negative control. Plates were incubated at 15 °C for 48 h, then the number of the survived larvae was counted. All experiments were performed in triplicate. The LC_50_ value was calculated by the same way descrived in Section 3.5.

### 3.8. Bangia fuscopurpurea Assay

The filamentous red-macroalga, *Bangia fuscopurpurea*, was suspended in 24-well plates containing 792 μL of artificial sea water, then 8 μL of compound **1** (2.000, 1.000, 0.500, 0.250, 0.125, 0.625 mg/mL in DMSO) was added, then incubated at 15 °C for 4 days with 14 h light–10 h dark photocycle. Pure DMSO (16 μL) was used as a negative control. The cells of *B. fuscopurpurea* were dyed by soaking in erythrosine B solution (0.1 mg/mL in artificial sea water), and number of the pink colored dead cells and the green colored live cells were counted under microscope. Experiments were done in triplicate. The LC_50_ and 95% confidence interval values were calculated by the same way descrived in Section 3.5.

### 3.9. Oryzias sp. Assay

After taming *Oryzias* sp. (Medaka fish) in plastic containers for 2 days at 20 °C, 10 individuals were transferred into each test tank containing 1.0 L water. Compound **1** (1.6 mg/mL in DMSO) was added at the final concentrations of 1.6, 0.16, and 0.016 μg/mL, respectively. Tanks were kept at 20 °C and observed for 48 hours, and number of live fish was counted. This experiment was performed once.

### 3.10. Co-Cultivation Assay

The colony of *Alteromonas* sp. D on an agar plate was picked by sterilized needle and inoculated in the early stationary phase *C. antiqua* culture (3 × 10^4^ cells/mL, 20 mL modified SWM-3 medium in 100 mL Erlenmeyer flasks), then incubated for 2 days under the same culture conditions described above. Then, whole medium was transferred to the centrifuge bottles and separated to cell pellet and supernatant by centrifugation. The resulting cell pellet was extracted with EtOH (10 mL). Supernatant was subjected to solid phase extraction by Sep-Pak C18 short columns (Waters, Milford, MA, USA). Short columns were washed with distilled water (5 mL), and eluted by MeOH (10 mL). Both cell pellet and supernatant extracts were combined, dried *in vacuo*, and re-dissolved in MeOH (10 mg/mL) to prepare LC-MS samples. A culture of *C. antiqua* with the same condition except for absence of *Alteromonas* sp. D was used as a negative control. All experiments were performed in duplicate.

### 3.11. LC-MS Quantiative Analysis

The samples prepared above were analyzed by LC-MS in multiple-reaction-monitoring (MRM) mode using the following conditions; column: Develosil RPAQUEOUS (2.0 × 100 mm, Nomura Chemical); flow rate: 0.2 mL/min; solvents: 30% aqueous MeOH to 100% MeOH containing 0.2% AcOH; MRM condition: precursor ion *m/z* 213.00 (M+H)^+^, daughter ion *m/z* 184.95 (M + H)^+^, collision energy –20 eV. Peak area was measured using the LC-solutions software (Shimadzu). Concentration of questiomycin A (**1**) in each sample was determined from the standard curve prepared from purified **1** as an authentic material.

### 3.12. Membrane Filter Separated Cultivation Assay

The cells of *C. antiqua* at early stationary phase were transferred to 6-well plates (1.3 × 10^4^ cells/mL, 7 mL/well), then wells of one group (group B) were covered by TC-insert filters (pore size 0.4 μm, Sarstedt, Tokyo, Japan) to separate inner and outer wells. After overnight incubation at 20 °C using a 14:10 h light–dark photocycle, the culture of *Alteromonas* sp. D (25 °C overnight in ST-medium, 1.4 μL) was inoculated in the wells of group A and inner wells of group B, then kept for 7 days. After evaluation of the cell morphorogy in each well under microscopic observation, conditioned media were recovered and extracted in the same manner described above, and re-dissolved in MeOH (200 μL) for the LC-MS analysis. All tests were performed in triplicate.

## 4. Conclusions

In the present study, questiomycin A (1-aminophenoxazine-3-one, (**1**)) and three new derivatives **2–4** were identified from the culture of the algicidal bacterium, *Alteromonas* sp. D, and compound **1–4** were shown to have potent algicidal activity. Compound **1** was also produced even in an oligotrophic medium, only when co-cultivated with *C. antiqua*. These observations suggested that the algicidal bacterium, *Alteromonas* sp. D, require close contact to prey microalgae to initiate biosynthesis of questiomycins in the oligotrophic environment. Local accumulation of the algicidal compound may trigger cell lysis although detailed mechanism is yet to be investigated. The contact-initiated production mechanism proposed here would be beneficial for algicidal bacteria to lyse algal cells using minimal production of secondary metabolites, and components in the lyzed cell can be utilized by bacterium. Further understanding of chemical and ecological processes between algicidal bacteria and blooming algae, especially in natural environment, is necessary to utilize this ecologically friendly strategy for preventing HAB.

## Figures and Tables

**Table 1:** ^1^H NMR data for **1**–**4** (*δ*_H_, mult. (*J* in Hz), DMSO-*d*_6_).

	1	2	3	4
1	6.36, s			
4	6.35, s	6.46, s	6.39, s	6.40, s
7	7.49, d (7.8)	7.51, m	7.51, m	7.53, m
8	7.46, t (7.9)	7.51, m	7.51, m	7.53, m
9	7.39, t (7.5)	7.41, m	7.43, m	7.44, m
10	7.70, d (7.9)	7.70, d (7.8)	7.82, d (7.9)	7.80, d (7.9)
NH_2_	6.60, brs	7.31, brs	6.95, brs	6.98, brs
		8.46, brs		
SOCH_3_		2.98, s		
SCH_3_			2.31, s	

**Table 2:** ^13^C NMR data for **1**–**4** (*δ*_C_, DMSO-*d*_6_).

	1	2	3	4
1	98.3	104.5	102.1	94.7
2	148.8	148.8	146.5	149.5
3	180.2	178.2	178.4	177.4
4	103.4	103.6	103.3	102.4
5	148.2	147.4	148.8	145.6
6	141.9	142.1	141.8	142.1
7	115.9	116.0	115.7	115.8
8	128.8	126.7	129.4	129.9
9	125.2	125.5	125.4	125.5
10	127.9	128.0	128.5	128.4
11	133.7	132.5	133.3	133.1
12	147.3	144.5	150.1	143.9
SOCH_3_		39.0		
SCH_3_			17.1	

**Table 3:** LC_50_ and 95% confidence interval values of questiomycins against selected microalgae and aquatic organisms (μM).

Organisms	1	2	3	4
*Chattonella antiqua*	0.64 ± 0.19 ^a^	0.18 ± 0.25 ^a^	6.37 ^b^	0.20 ± 0.04 ^a^
*Karenia mikimotoi*	1.18 ^c^			
*Chaetoceros didymus*	0.59 ^c^			
*Bangia fuscopurpurea*	25.9 ± 1.32 ^a^			
*Artemia salina*	91.7 ^b^			> 68.7 ^d^
*Oryzias* sp.	0.75-7.5 ^e^			

^a^ LC_50_ ± 95% confidence interval (CI). ^b^ 95% CI was not calculatable due to 100% lethal was not observed within the concentration tested. ^c^ Concentration at which more than 90% algal cells lysed. d More than half of indibiduals survived at the highest concentration tested. ^e^ 10/10 and 0/10 fishes survived for 48 h at 0.75 and 7.5 μM, respectively.

## Supplementary Materials

The following materials are available online at https://www.mdpi.com/1420-3049/24/24/4522/s1, Figure S1: ^1^H NMR spectrum of **1** (DMSO-*d*_6_), Figure S2: ^13^C NMR spectrum of **1** (DMSO-*d*_6_), Figure S3: COSY spectrum of **1** (DMSO-*d*_6_), Figure S4: HSQC spectrum of **1** (DMSO-*d*_6_), Figure S5: HMBC spectrum of **1** (DMSO-*d*_6_), Figure S6: ^1^H NMR spectrum of **2** (DMSO-*d*_6_), Figure S7: ^13^C NMR spectrum of **2** (DMSO-*d*_6_), Figure S8: COSY spectrum of **2** (DMSO-*d*_6_), Figure S9: HSQC spectrum of **2** (DMSO-*d*_6_), Figure S10: HMBC spectrum of **2** (DMSO-*d*_6_), Figure S11: NOESY spectrum of **2** (DMSO-*d*_6_), Figure S12: ^1^H NMR spectrum of **3** (DMSO-*d*_6_), Figure S13: ^13^C NMR spectrum of **3** (DMSO-*d*_6_), Figure S14: COSY spectrum of **3** (DMSO-*d*_6_), Figure S15: HSQC spectrum of **3** (DMSO-*d*_6_), Figure S16: HMBC spectrum of **3** (DMSO-*d*_6_), Figure S17: NOESY spectrum of **3** (DMSO-*d*_6_), Figure S18: ^1^H NMR spectrum of **4** (DMSO-*d*_6_), Figure S19: ^13^C NMR spectrum of **4** (DMSO-*d*_6_), Figure S20: COSY spectrum of **4** (DMSO-*d*_6_), Figure S21: HSQC spectrum of **4** (DMSO-*d*_6_), Figure S22: HMBC spectrum of **4** (DMSO-*d*_6_), Figure S23: LCMS chromatogram of *Alteromonas* sp. D culture broth supplemented with *o*-aminohenol and dimethylsulfide, Figure S24: LCMS chromatogram of *Alteromonas* sp. D culture broth supplemented with *o*-aminohenol and dimethyldisulfide, Figure S25: Cell morphology of live and dead cells of *C. antiqua*, Figure S26: Results of quantitative algicidal assay of questiomycins, Figure S27: Morphology of *K. mikimotoi* and *C. didymus* treated with lethal does of **1**, Figure S28: Morphological change of selected microalgae over questiomycin A (**1**), Figure S29: Picture of *C. antiqua* culture co-cultivation with or without *Alteromonas* sp. D, Figure S30: LCMS chromatograms of the extract of co-culture (A) and separate-culture (B) in 6 well plates, Table S1: Literature NMR data of compound **1**, Table S2: Recipe of the modified SWM-3 medium, Table S3: method and results of questiomycin A (**1**) plate absorption test. Raw NMR data (FID) are available at https://www.mdpi.com/1420-3049/24/24/4522/s2.
