# Supplementary material for: Questiomycins, Algicidal Compounds Produced by the Marine Bacterium Alteromonas sp. D and their Production Cue
Source: Molecules. 2019 Dec 10;24(24):4522. doi: 10.3390/molecules24244522 (PMC6943571; doi:10.3390/molecules24244522)
Supplement: Supplementary file 1 [file molecules-24-04522-s001.zip › molecules-630540 suppl from proof.pdf]

Supplementary Materials

Questiomycins, algicidal compounds produced by the marine bacterium *Alteromonas* sp. D and their production mechanism

Saki Umetsu[1], Mamoru Kanda[1], Ichiro Imai[1,2], Ryuichi Sakai[1], Masaki J. Fujita[1,*]

[1]Graduate School of Fisheries Sciences, Hokkaido University, 3-1-1 Minato-cho, Hakodate, Hokkaido, 041-8611, Japan

[2] Lake Biwa Museum, 1091 Oroshimo-cho, Kusatsu, Shiga, 525-0001, Japan

*corresponding author at: Graduate School of Fisheries Sciences, Hokkaido University 3-1-1 Minato-cho, Hakodate, Hokkaido, 041-8611, Japan.

E-mail address: masakifujita@fish.hokudai.ac.jp (M.J. Fujita)

Figure S1: ^{1}H NMR spectrum of **1** with partially enlarged inset (DMSO-d_6).

Figure S2: ^{13}C NMR spectrum of **1** (DMSO-d_6).

Figure S3: COSY spectrum of **1** (DMSO-d_6).

Figure S4: HSQC spectrum of **1** (DMSO-d_6).

Figure S5: HMBC spectrum of **1** (DMSO-d_6).

Figure S6: ^{1}H NMR spectrum of **2** with partially enlarged inset (DMSO-d_6).

Figure S7: ^{13}C NMR spectrum of **2** (DMSO-d_6).

Figure S8: COSY spectrum of **2** (DMSO-d_6).

Figure S9: HSQC spectrum of **2** (DMSO-d_6).

Figure S10: HMBC spectrum of **2** (DMSO-d_6).

Figure S11: NOESY spectrum of **2** (DMSO-d_6).

Figure S12: ^{1}H NMR spectrum of **3** with partially enlarged inset (DMSO-d_6).

Figure S13: ^{13}C NMR spectrum of **3** (DMSO-d_6).

Figure S14: COSY spectrum of **3** (DMSO-d_6).

Figure S15: HSQC spectrum of **3** (DMSO-d_6).

Figure S16: HMBC spectrum of **3** (DMSO-d_6).

Figure S17: NOESY spectrum of **3** (DMSO-d_6).

Figure S18: ^{1}H NMR spectrum of **4** with partially enlarged inset (DMSO-d_6).

Figure S19: ^{13}C NMR spectrum of **4** (DMSO-d_6).

Figure S20: COSY spectrum of **4** (DMSO-d_6).

Figure S21: HSQC spectrum of **4** (DMSO-d_6).

Figure S22: HMBC spectrum of **4** (DMSO-d_6).

Figure S23: LCMS chromatogram of *Alteromonas* sp. D culture broth supplemented with *o*-aminohenol and dimethylsulfide.

Figure S24: LCMS chromatogram of *Alteromonas* sp. D culture broth supplemented with *o*-aminohenol and dimethyldisulfide.

Figure S25: Cell morphology of live and dead cells of *C. antiqua.*

Figure S26: Algicidal assay results of questiomycins against *C. antiqua*.

Figure S27: Morphological change of *K. mikimotoi and C. didymus* cells caused by addition of questiomycin A.

Figure S28: Acute toxicity assay results of the macroalga, *B. fuscopurpurea* and the crustacean, *A. salina*, treated with serial dilution of questiomycins.

Figure S29: Picture of conditioned media of *C. antiqua* and *Alteromonas* sp. D co-cultivation, and *C. antiqua* without *Alteromonas* sp. D.

Figure S30: LCMS chromatograms of the extracts of co-culture and separate-culture of *C. antiqua* and *Alteromonas* sp. D.

Table S1: Literature NMR data of compound **1**.

Table S2: Recipe of the modified SWM-3 medium.

Table S3: Method and result of questiomycin A (**1**) absorption test.

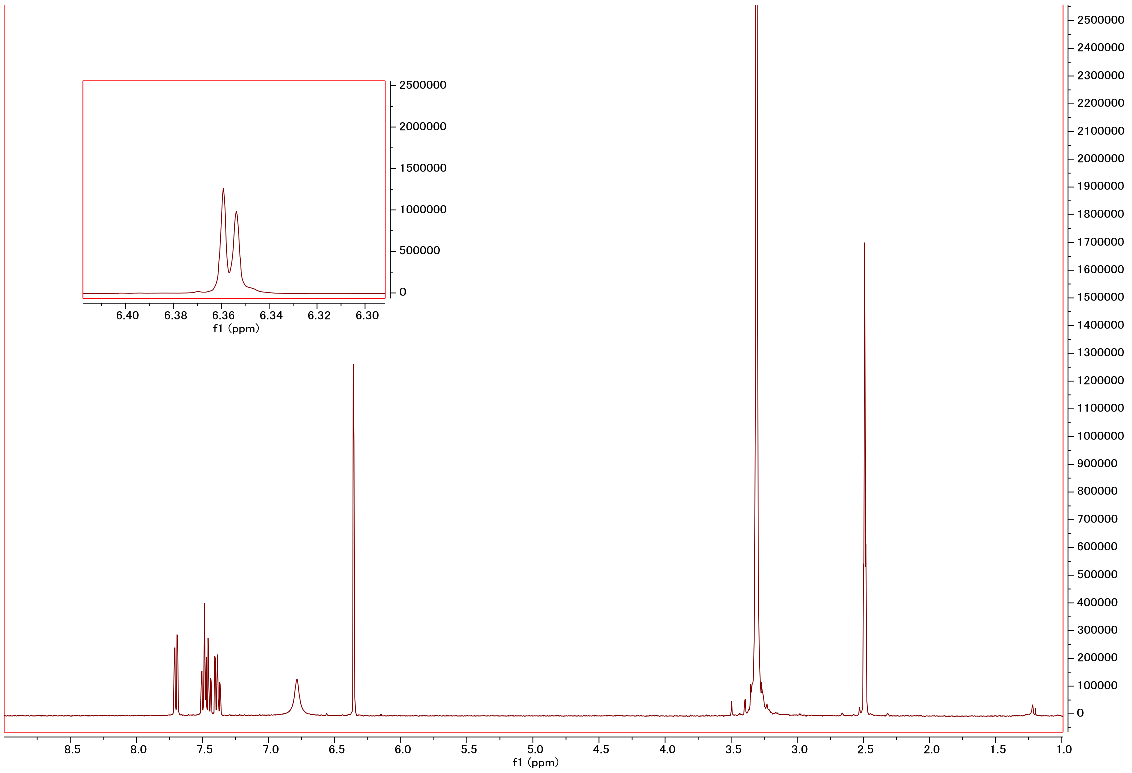

Figure S1. ^{1}H NMR spectrum of questiomycin A (**1**) in DMSO-*d6*. Inset is an expansion of signal H-1 and 4 region (δ_H 6.25-6.50).

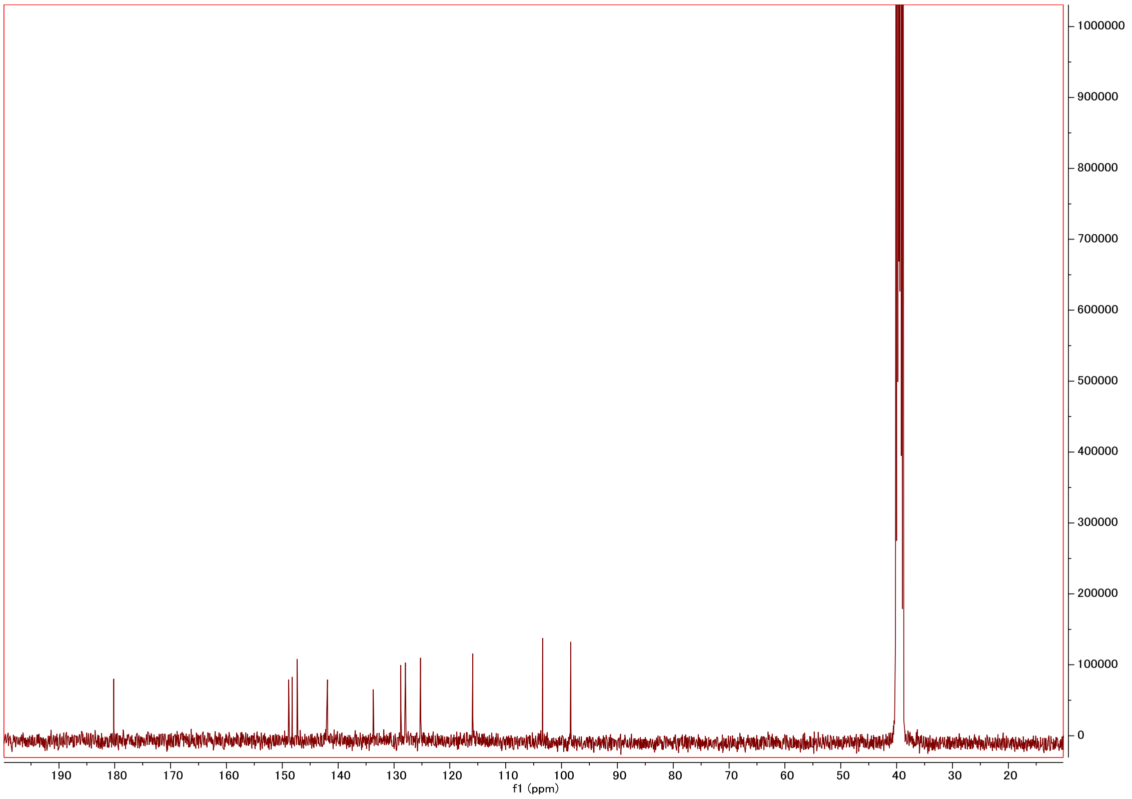

Figure S2. ^{13}C NMR spectrum of questiomycin A (**1**) in DMSO-*d6*.

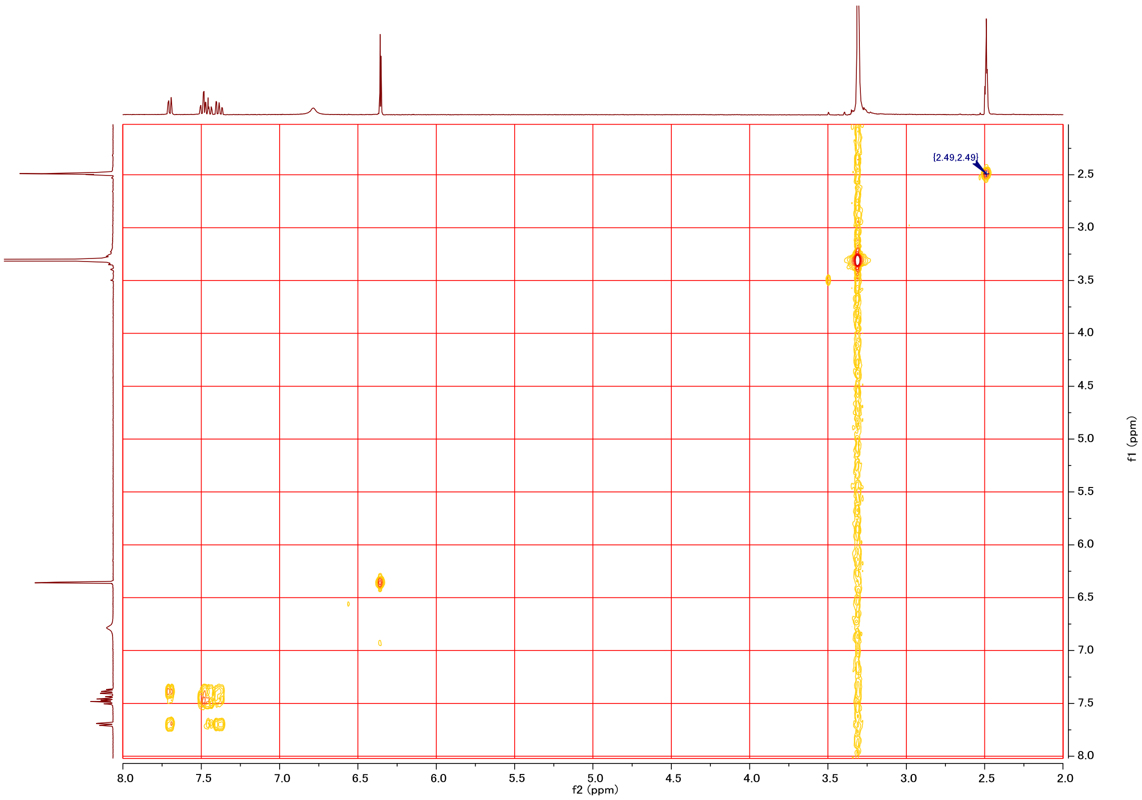

Figure S3. COSY spectrum of questiomycin A (**1**) in DMSO-*d6*.

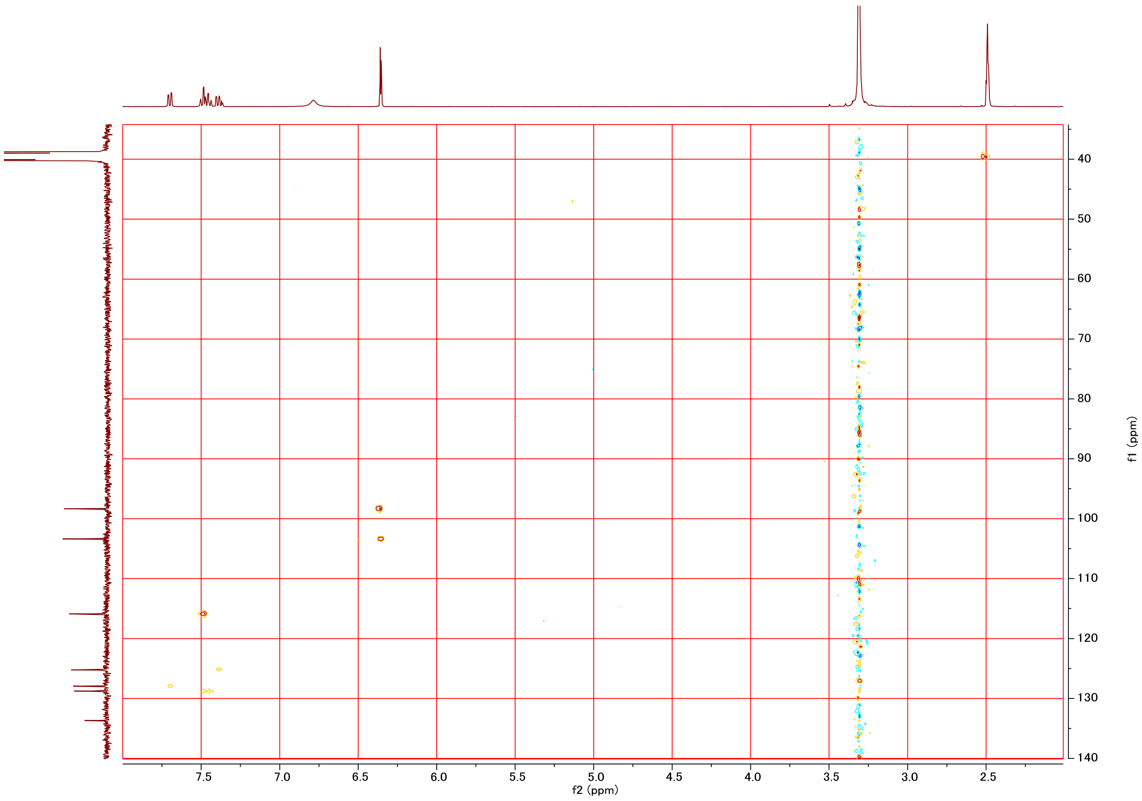

Figure S4. HSQC spectrum of questiomycin A (**1**) in DMSO-*d6*.

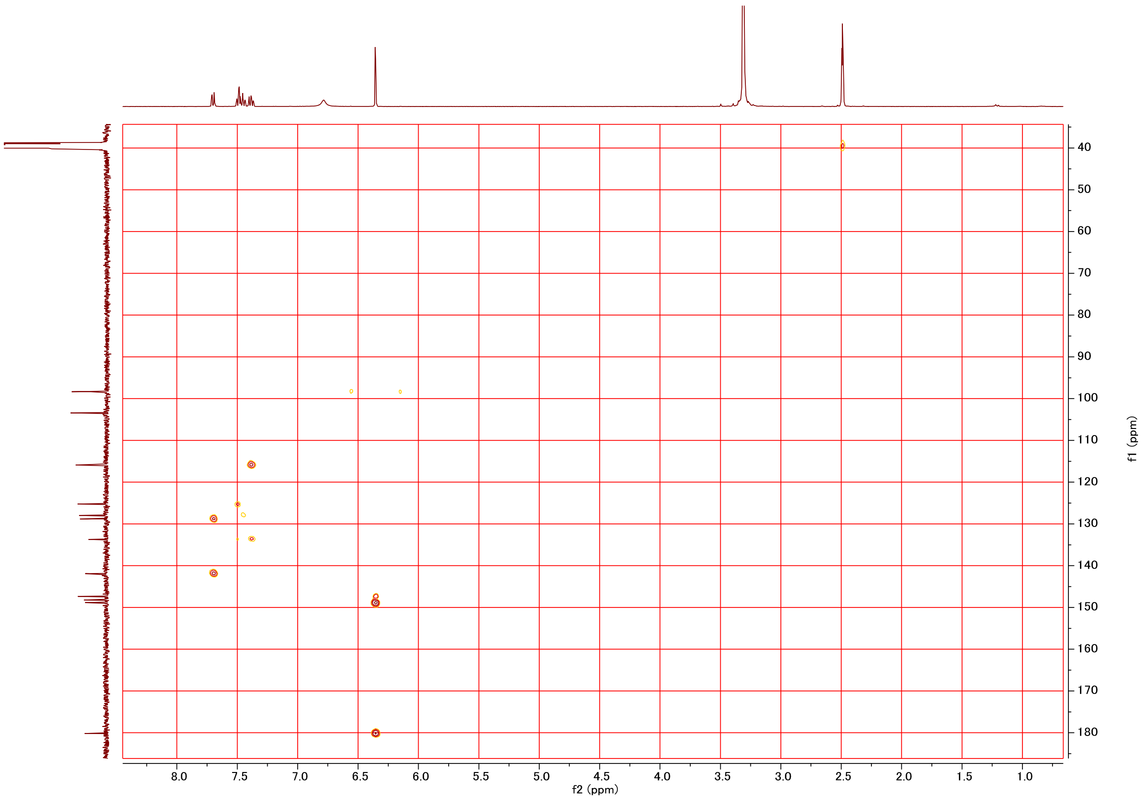

Figure S5. HMBC spectrum of questiomycin A (**1**) in DMSO-*d6*.

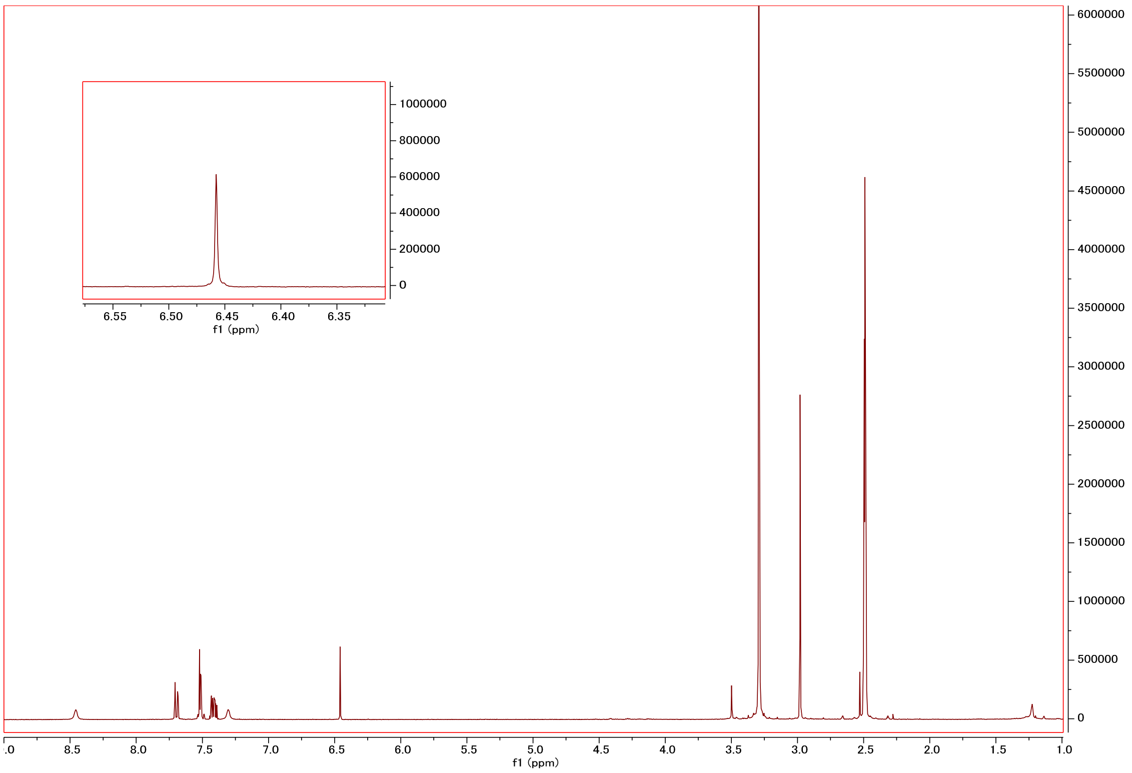

Figure S6. ^{1}H NMR spectrum of questiomycin C (**2**) in DMSO-*d6*. Inset is an expansion of signal H- 4 region (δ_H 6.25-6.60).

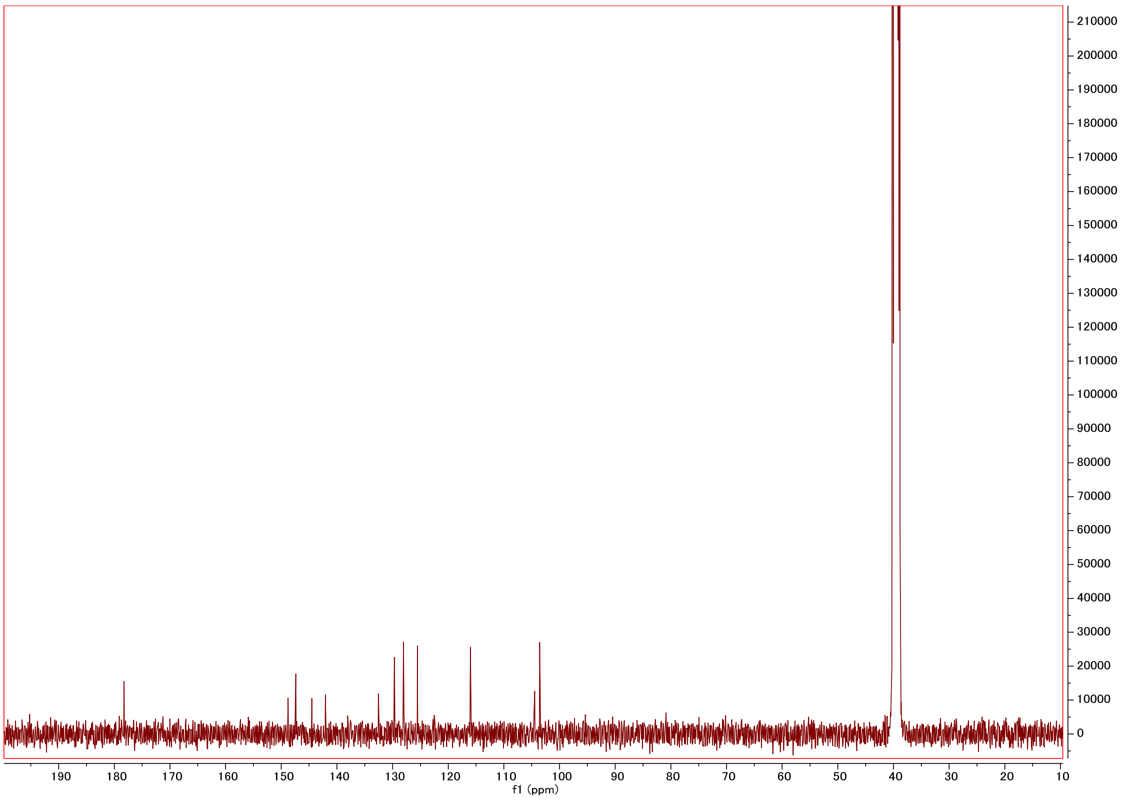

Figure S7. ^{13}C NMR spectrum of questiomycin C (**2**) in DMSO-*d6*.

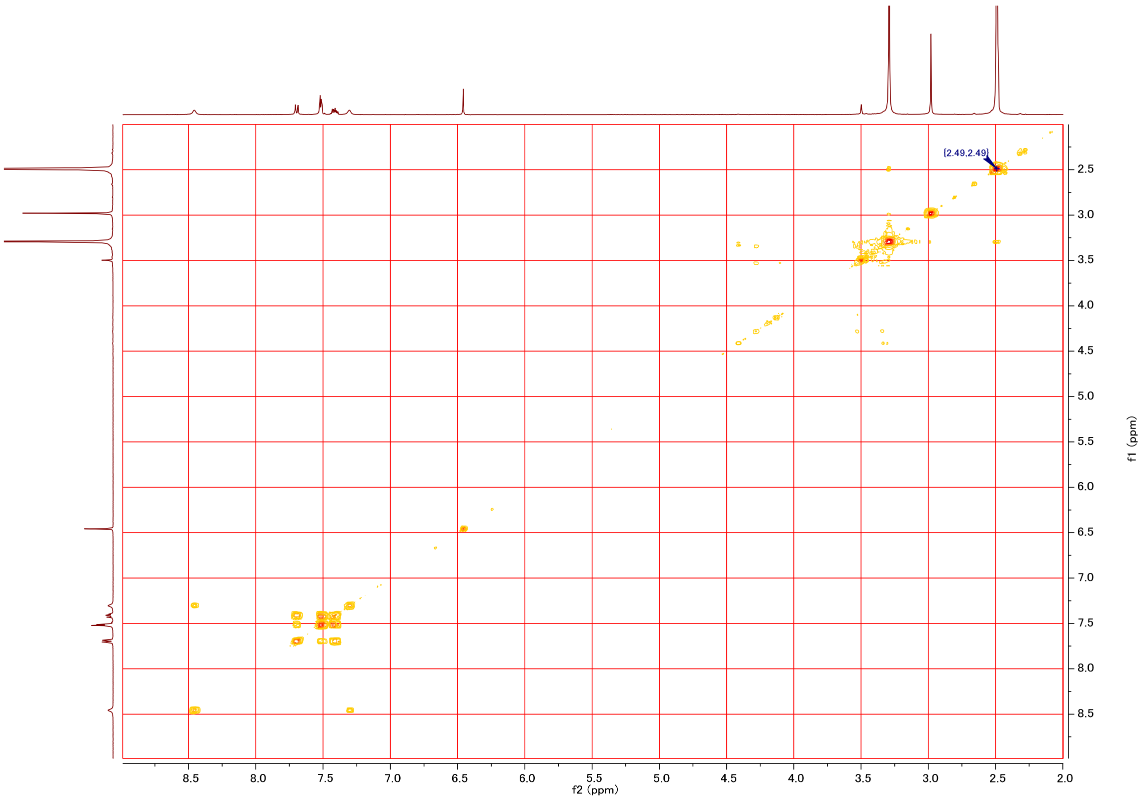

Figure S8. COSY spectrum of questiomycin C (**2**) in DMSO-*d6*.

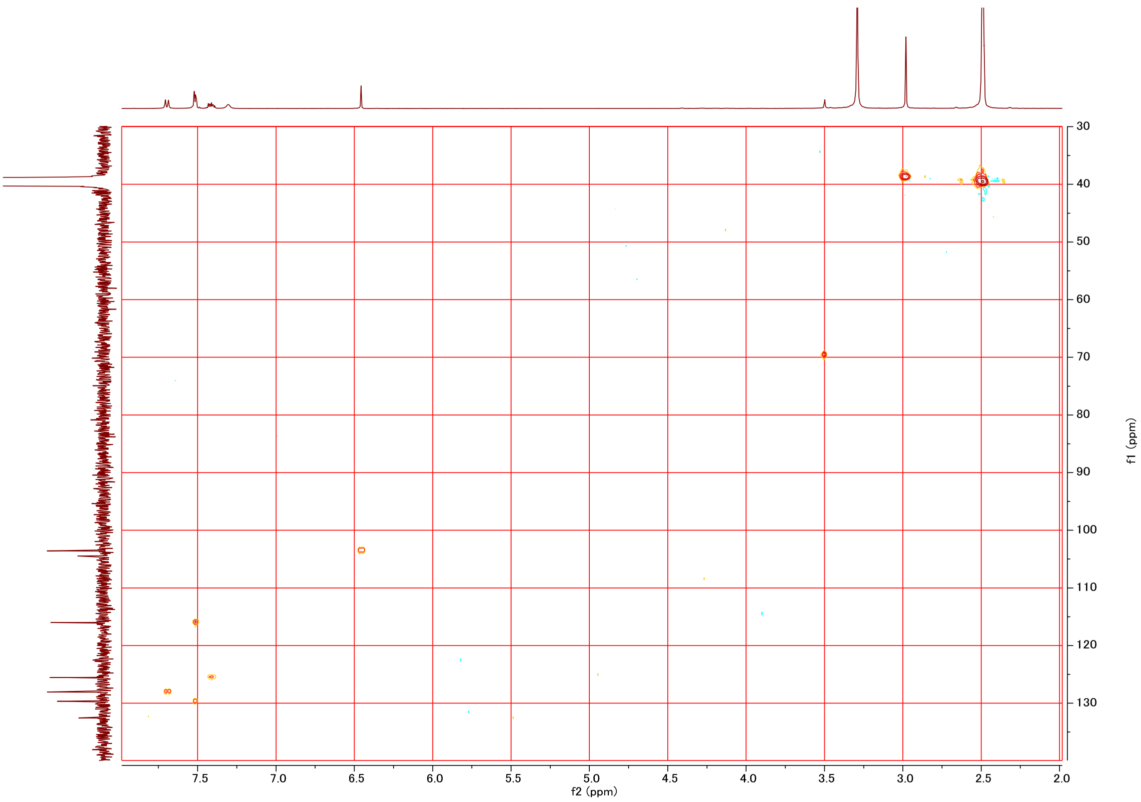

Figure S9. HSQC spectrum of questiomycin C (**2**) in DMSO-*d6*.

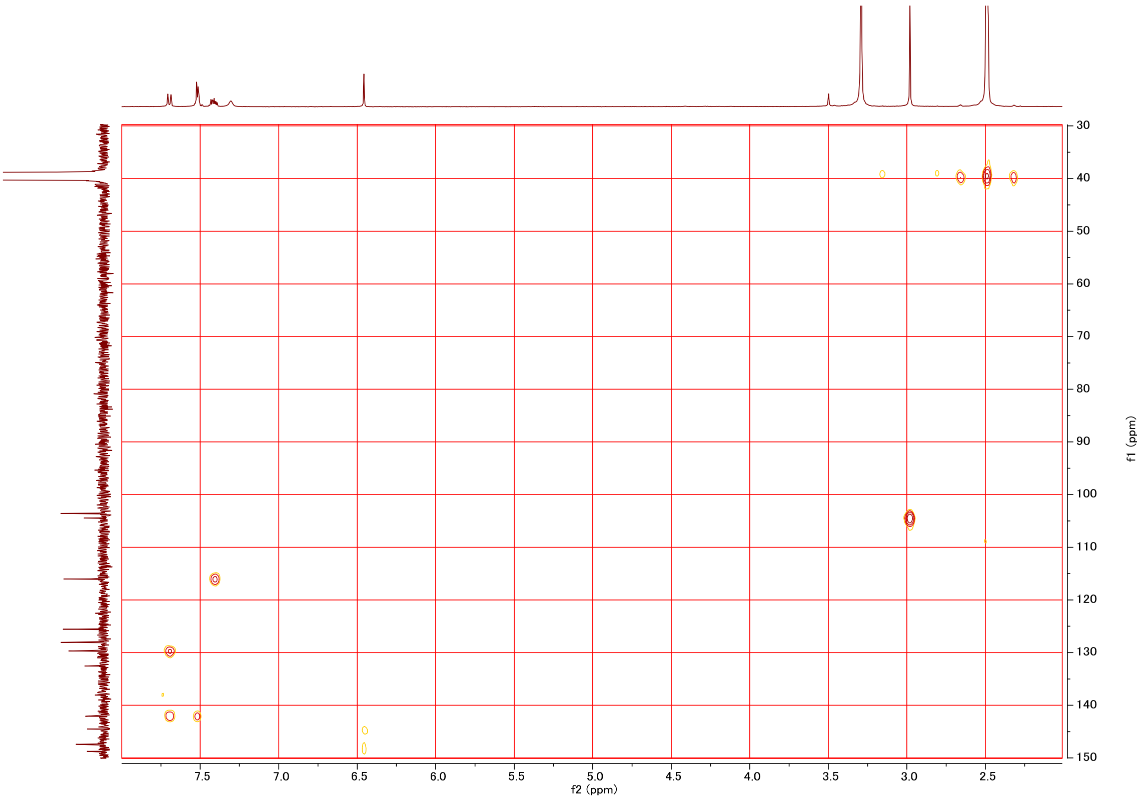

Figure S10. HMBC spectrum of questiomycin C (**2**) in DMSO-*d6*.

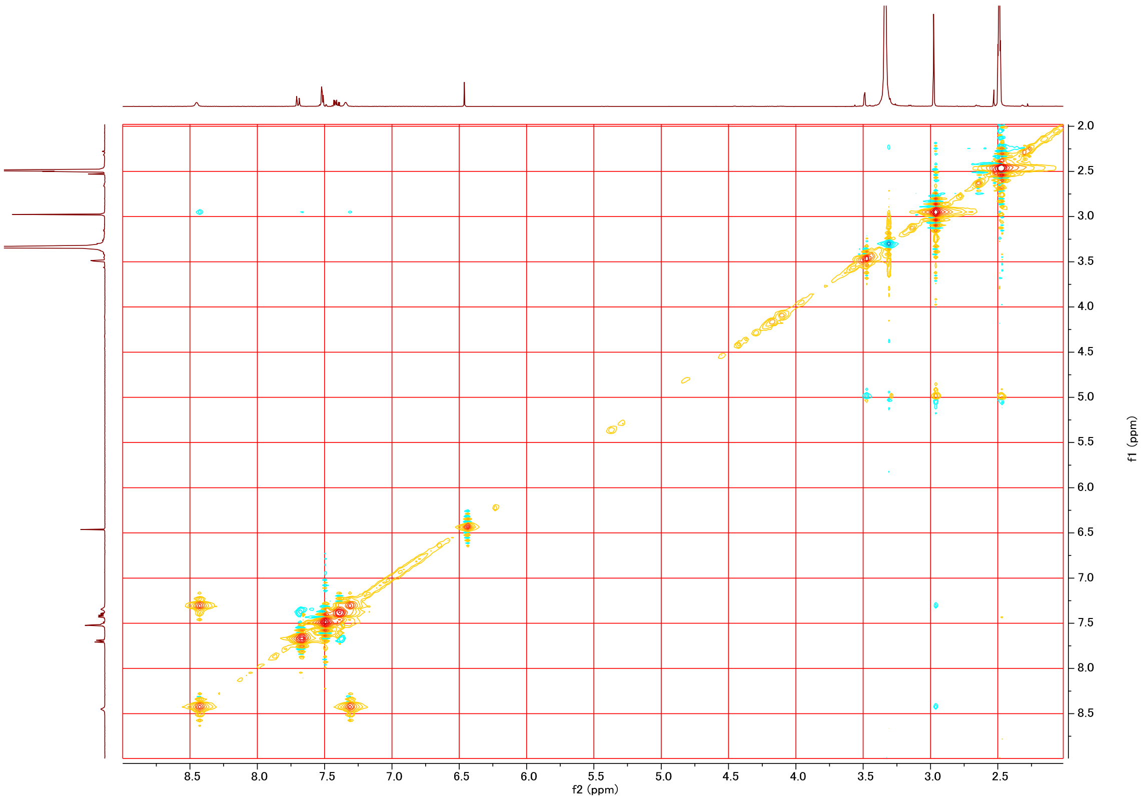

Figure S11. NOESY spectrum of questiomycin C (**2**) in DMSO-*d*6.

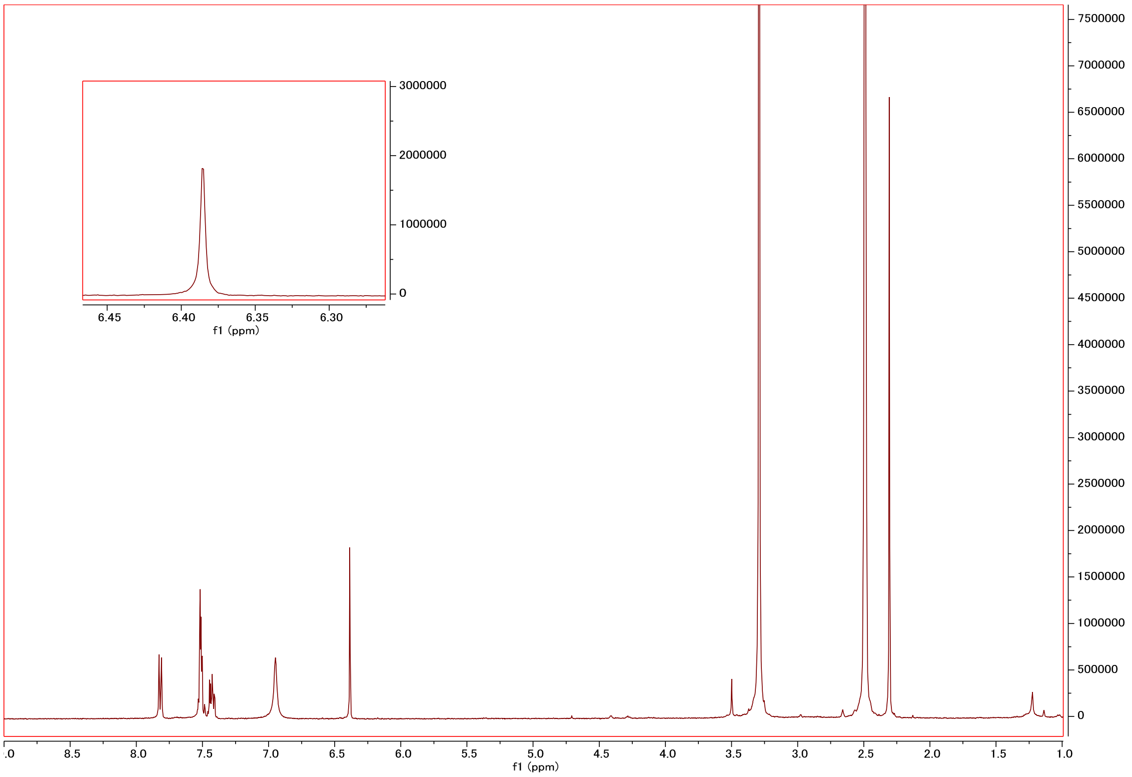

Figure S12. ^{1}H NMR spectrum of questiomycin D (**3**) in DMSO-*d*6. Inset is an expansion of signal H- 4 region (δ_H 6.25-6.50).

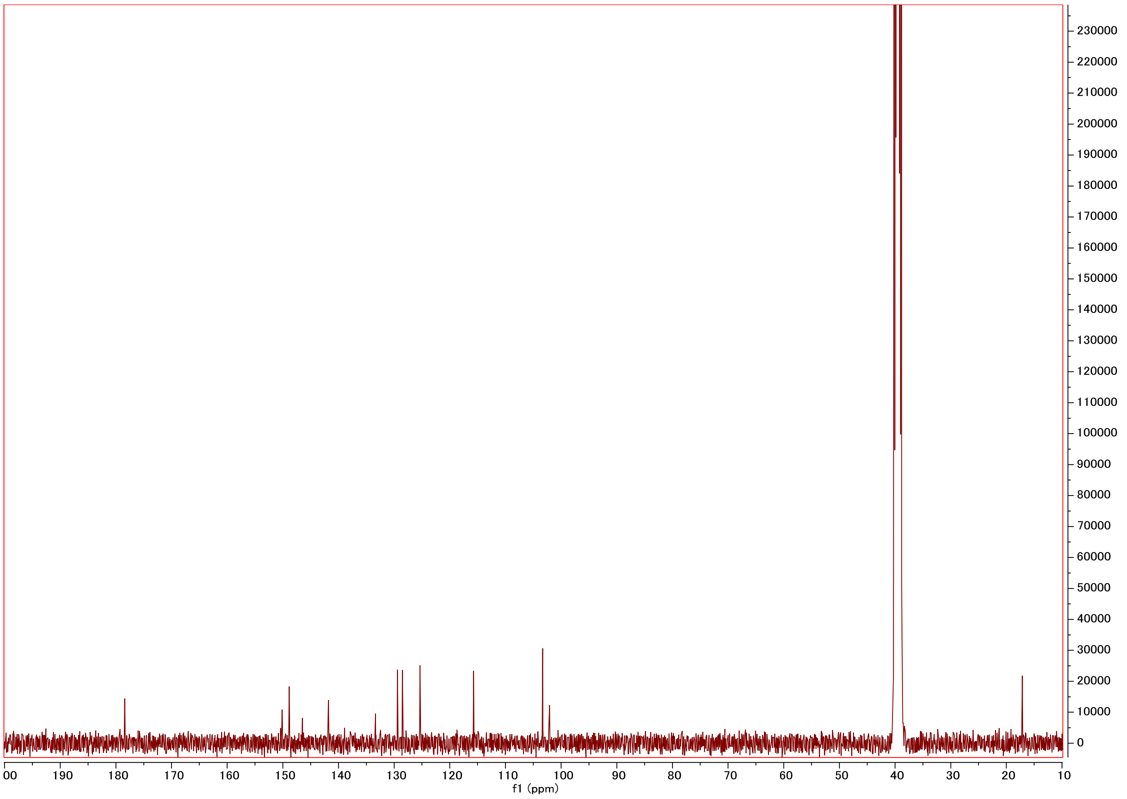

Figure S13. ^{13}C NMR spectrum of questiomycin D (**3**) in DMSO-*d6*.

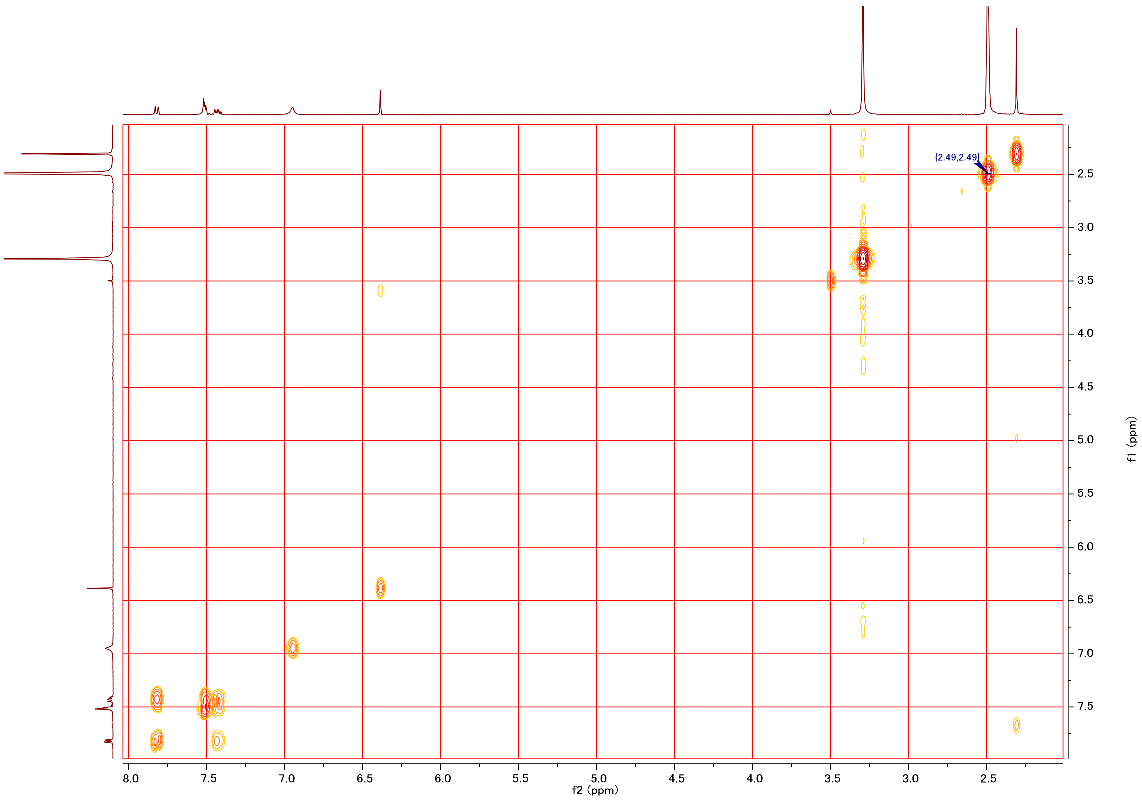

Figure S14. COSY spectrum of questiomycin D (**3**) in DMSO-*d6*.

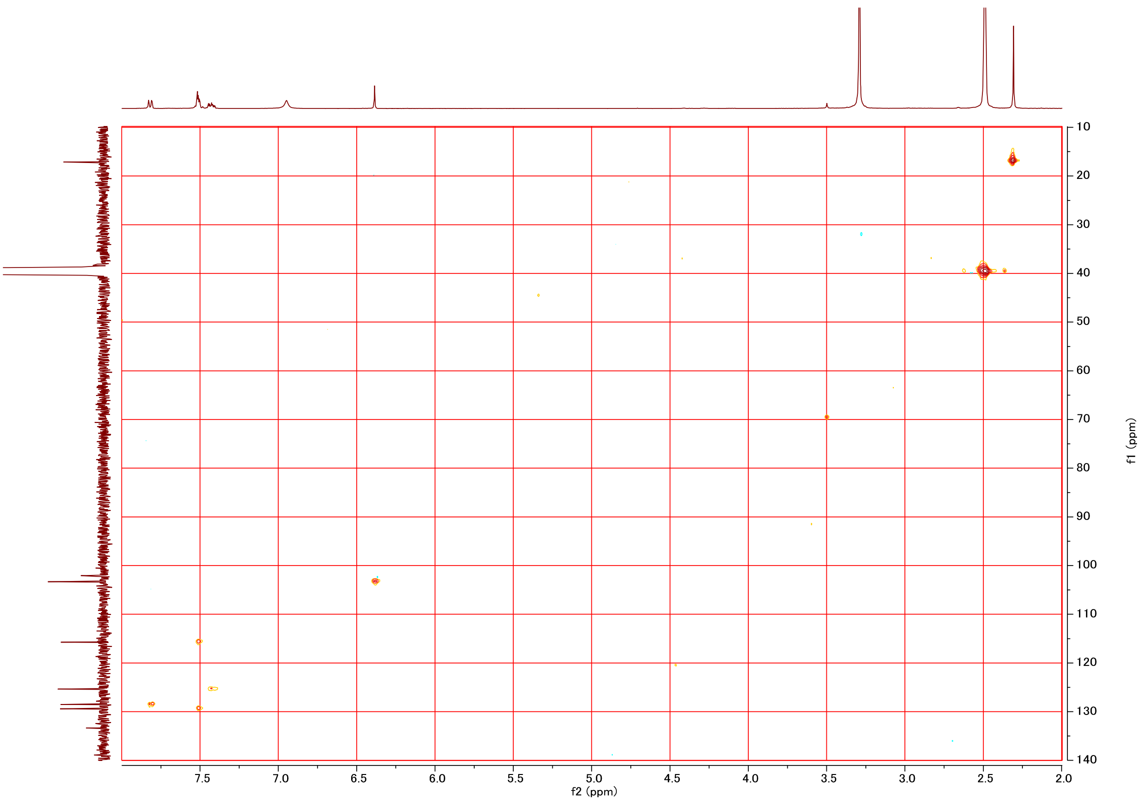

Figure S15. HSQC spectrum of questiomycin D (**3**) in DMSO-*d*6.

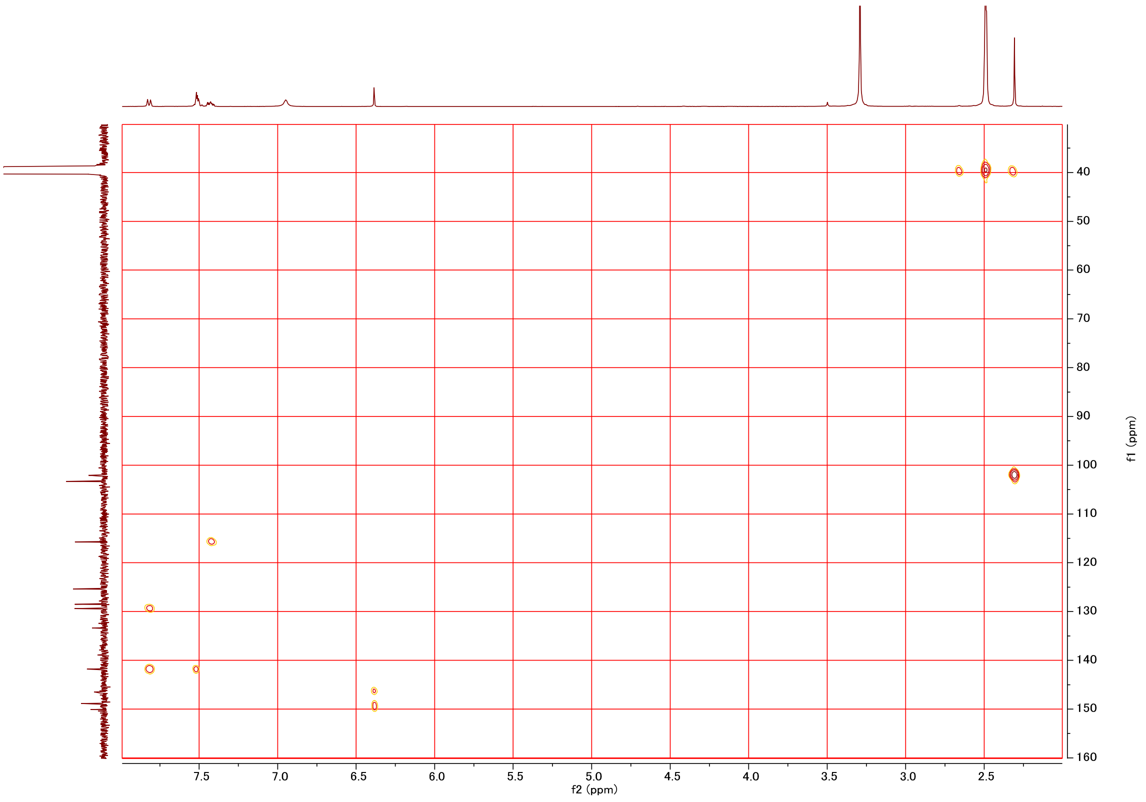

Figure S16. HMBC spectrum of questiomycin D (**3**) in DMSO-*d*6.

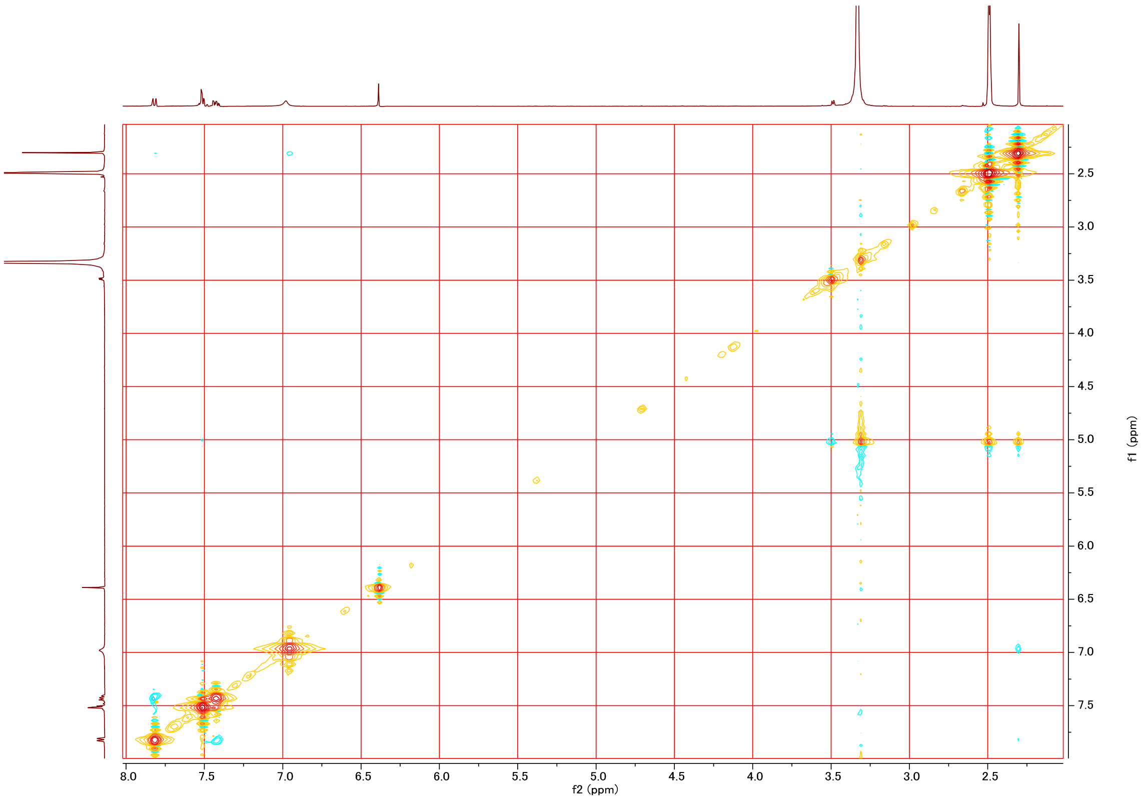

Figure S17. NOESY spectrum of questiomycin D (**3**) in DMSO-*d6*.

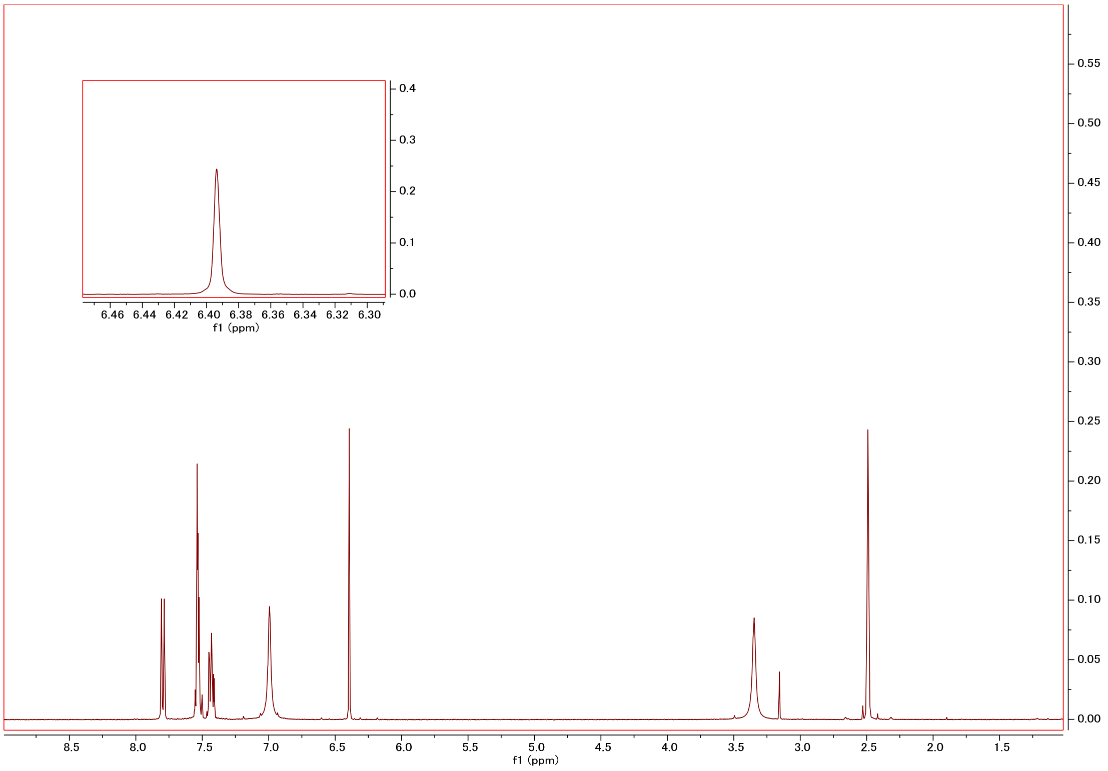

Figure S18. ^{1}H NMR spectrum of questiomycin E (**4**) in DMSO-*d6*. Inset is an expansion of signal H- 4 region (δ_H 6.25-6.50).

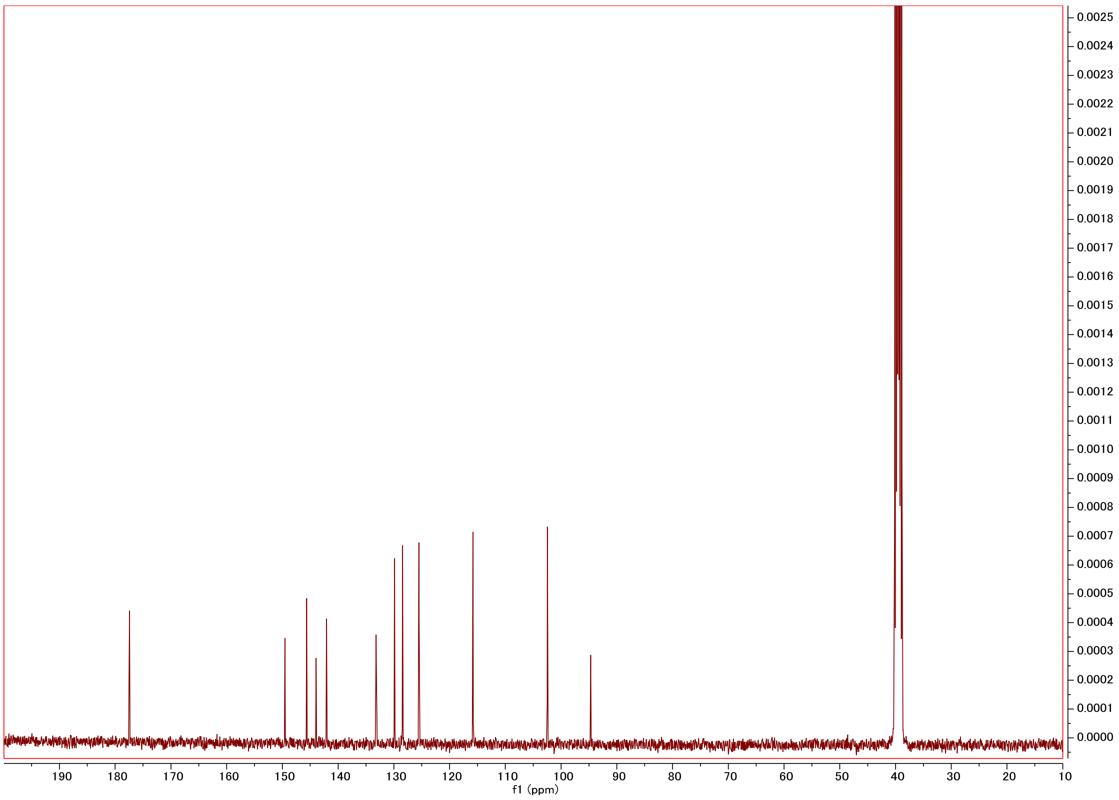

Figure S19. ^{13}C NMR spectrum of questiomycin E (**4**) in DMSO-*d6*.

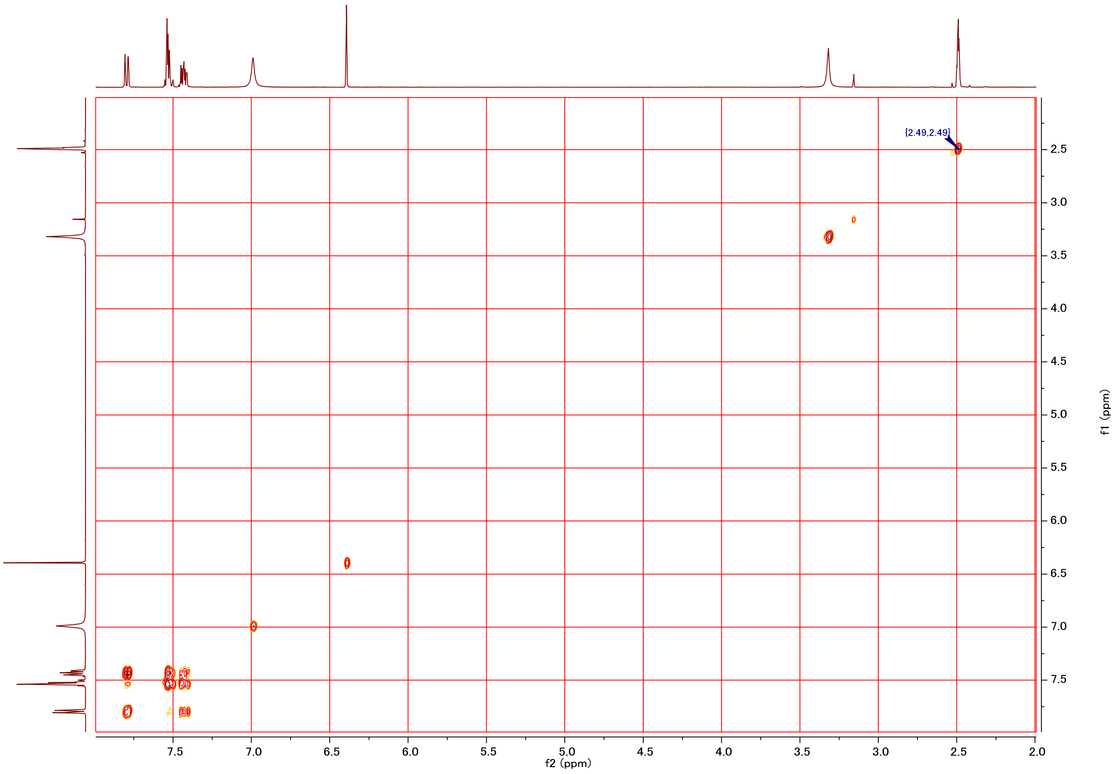

Figure S20. COSY spectrum of questiomycin E (**4**) in DMSO-*d6*.

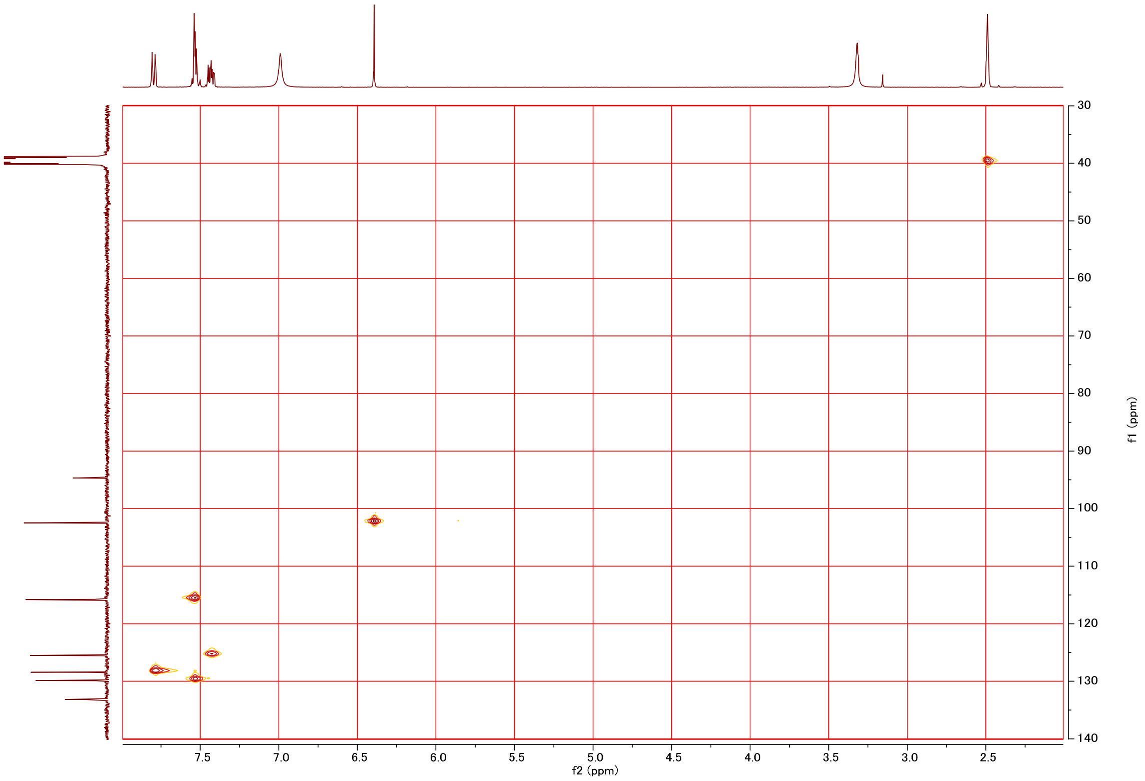

Figure S21. HSQC spectrum of questiomycin E (**4**) in DMSO-*d6*.

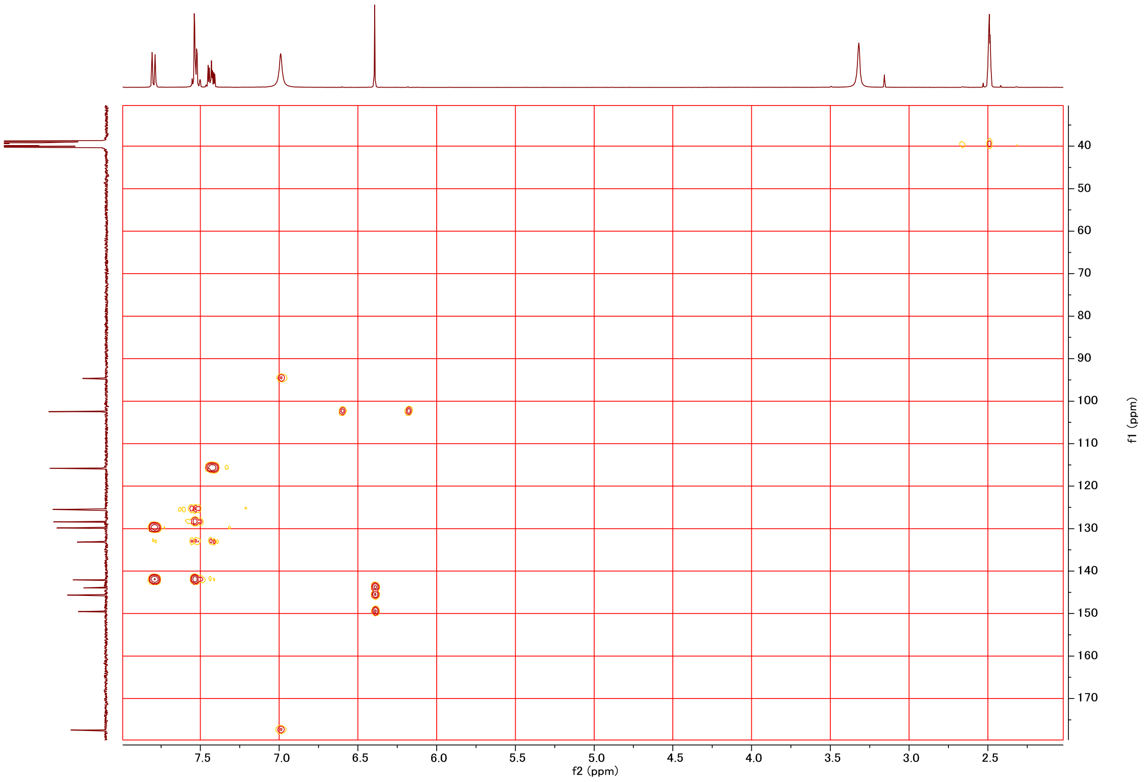

Figure S22. HMBC spectrum of questiomycin E (**4**) in DMSO-*d6*.

o-Aminophenol and Sulfur Sources Supplemented Fermentation

Filter-sterilized ethanol solution of *o*-aminophenol (final concentration 100 mg/L) and sulfur sources, either dimethylsulfide (500 μL/L) or dimethyldisulfide (500 μL/L), were added into *Alteromonas* sp. D overnight culture (10 mL of 1/3-ST medium; trypton 1.67 g/L, yeast extract 0.17 g/L in artificial sea water), then further shaken for four days at 30 ˚C (180 rpm). Centrifuge clarified conditioned media were dissolved in 50 times volume of MeOH, then subjected to the LC-MS analysis equipped with C30 column using scan-mode analysis (*m/z* 100-500). Only questiomycin A (**1**: *m/z* = 213.0) was observed in both cases (Figure S23 and S24).

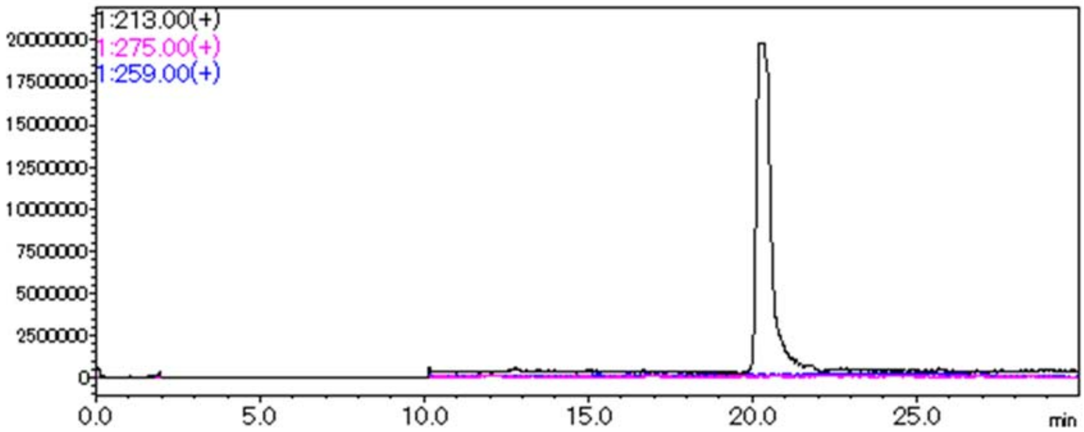

Figure S23. LCMS chromatogram of *Alteromonas* sp. D culture broth supplemented with *o*-aminohenol and dimethylsulfide.

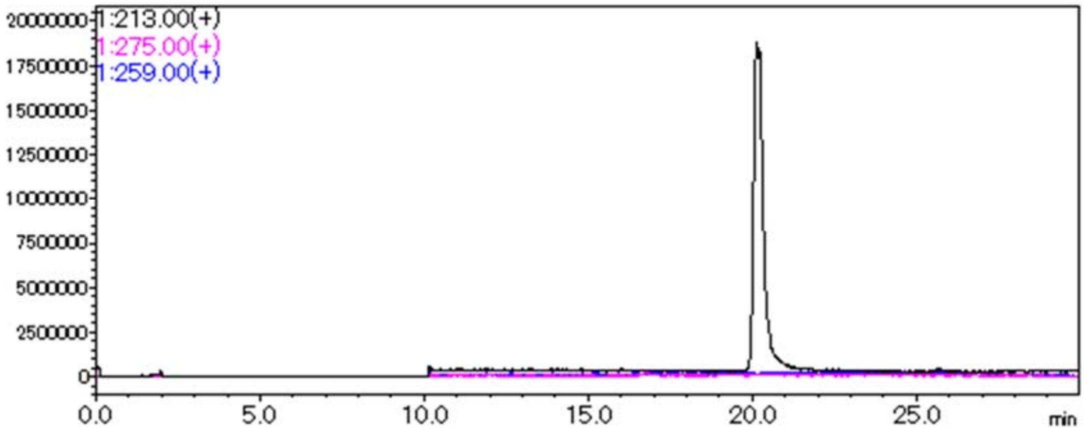

Figure S24. LCMS chromatogram of *Alteromonas* sp. D culture broth supplemented with *o*-aminohenol and dimethyldisulfide.

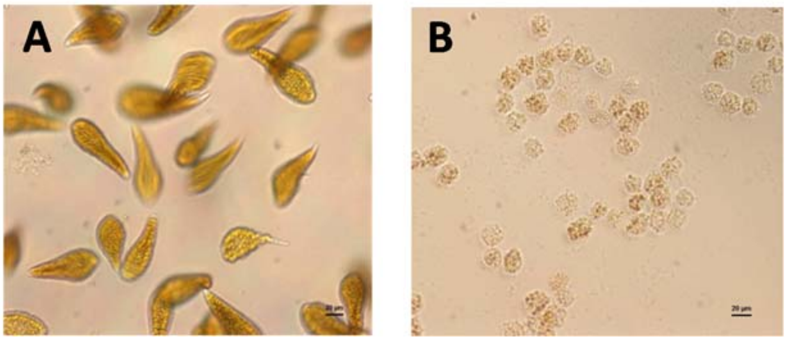

Figure S25. A: Cell morphology of live cells of *C. antiqua*. **B:** Dead cells of *C. antiqua* lysed by addition of compound **1**. Scale Bar: 20 μm.

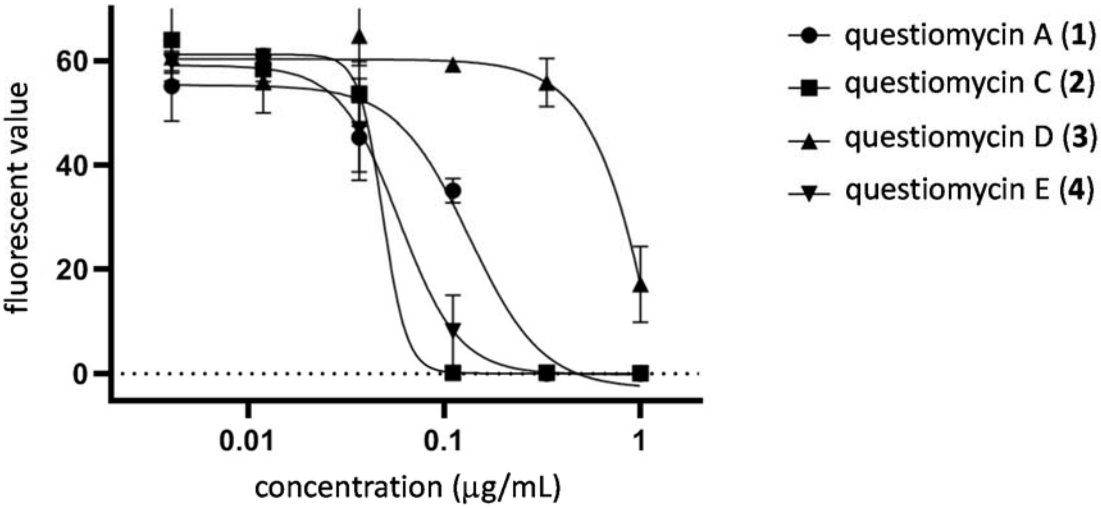

Figure S26. Algicidal activity of questiomycins (**1-4**) against *C. antiqua*. Cell density was estimated from autofluorescence of the live cells. Each data point represent average values ± standard deviation (n=3).

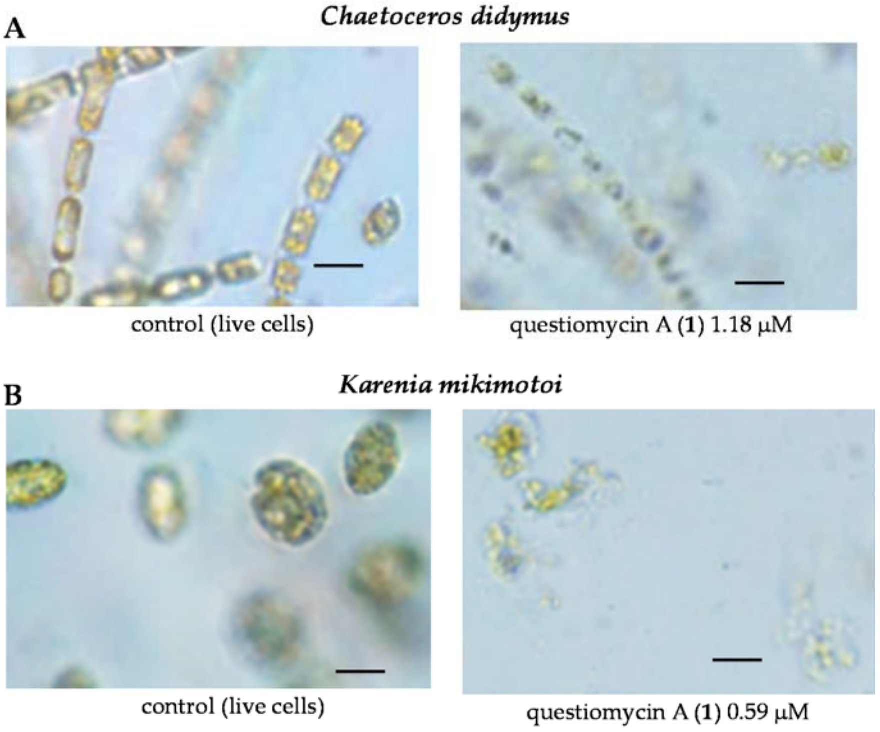

Figure S27. Representative morphological changes of *C. didymus* and *K. mikimotoi* cells caused by addition of questiomycin A (**1**) at the concentration indicated. In *C. didymus* and *K mikimotoi* cells, nearly all the cells after addition of **1** became fuzzy and cytosol shrinking or leakage were observed. Scale Bar: 20 μm.

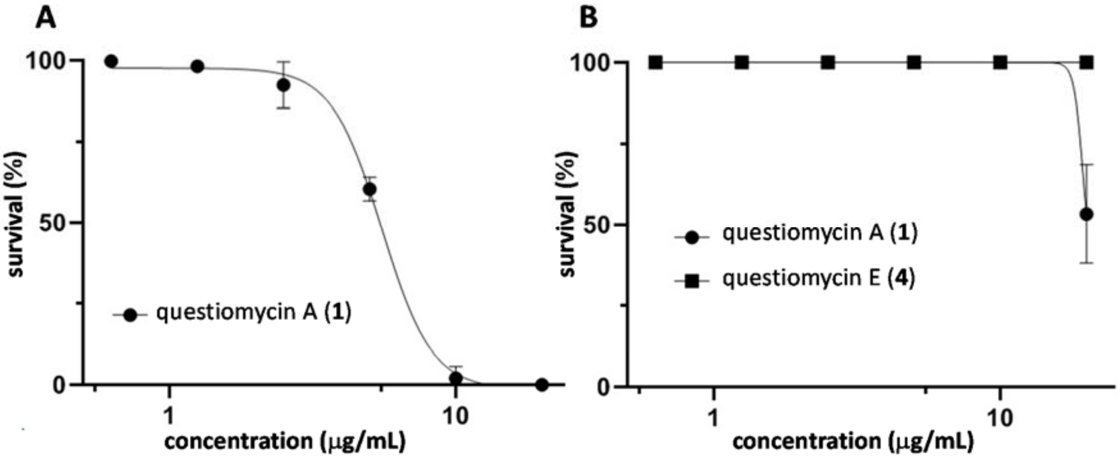

Figure S28. Acute toxicity assay results of the macroalga, *B. fuscopurpurea* (A) and the crustacean, *A. salina* (B), treated with serial dilution of questiomycins. Average values and standard deviation (n=3).

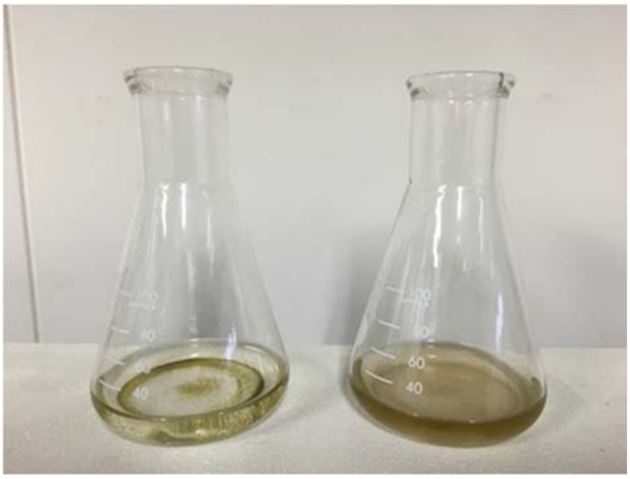

Figure S29. Conditioned media of *C. antiqua* and *Alteromonas* sp. D co-cultivation (left), and *C. antiqua* without *Alteromonas* sp. D (right). Debris of the lysed *C. antiqua* cells were deposited on the bottom of the flask to be clear medium (left). On the other hand, live algal cells were swarming in the medium to form water pigmentation (right).

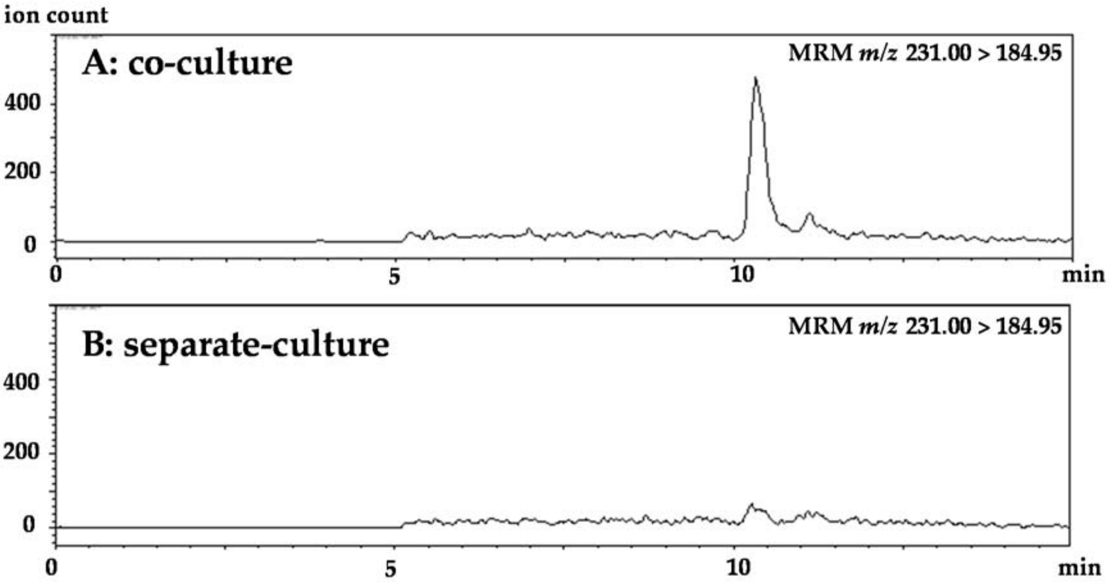

Figure S30. LCMS chromatograms of the extracts of co-culture (A) and separate-culture of *C. antiqua* and *Alteromonas* sp. D. in 6 well plates with and without separation membrane, respectively.

Table S1. NMR Data of **1**

	δ_H		δ_C	
	literature	experimental	literature	experimental
1	6.37, s	6.36, s	98.4	98.3
2			148.8	148.8
3			180.1	180.2
4	6.35, s	6.35, s	103.4	103.4
5			148.2	148.2
6			141.9	141.9
7	7.47, d (7.6)	7.47, m	115.8	115.9
8	7.45, m	7.47, m	128.7	128.8
9	7.38, d (7.6)	7.39, t (8.3)	125.2	125.2
10	7.70, d (7.6)	7.70, d (9.3)	127.9	127.9
11			133.7	133.7
12			148.8	147.3
NH2	6.73, brs	6.79, brs		

Table S2. Modified SWM-3 Medium

	$NaNO_3$	2mM
	$NaH_2PO4 \cdot 2H_2O$	0.1mM
	$Na_2SiO_3 \cdot 9H_2O$	0.2 mL
	Na_2-EDTA	30 μM
	Fe-EDTA	2 μM
	Na_2SeO_3	2 μM
	$Na_2MoO_4 \cdot 2H_2O$	100 nM
	TRIS	500 mg
	P-1 metals	10 mL
	S-3 vitamins	2 mL
	artificial seawater	1000 mL
	pH	7.7-7.8
P-1 metals		
	H_3BO_3	1 mmol
	$MnCl_2 \cdot 4H_2O$	3.5×10^{-2} mmol
	$ZnCl_2$	4.0×10^{-3} mmol
	$CoCl_2 \cdot 6H_2O$	1.0×10^{-4} mmol
	$CuCl_2 \cdot 2H_2O$	1.0×10^{-6} mmol
S-3 vitamins		
	vitamin B1-HCl	0.5 mg
	Ca-Pantothenate	0.1 mg
	nicotinic acid	0.1 mg
	p-aminobenzoic acid	10 μg
	biotin	1 μg
	inositol	5 mg
	folic acid	2 μg
	thymine	3 mg
	vitamin B12	1 μg

Table S3. Absorption Test of **1**

initial concentration (ng/mL)	3	30	300
polystylene plate (%)	94	79	80
glass vial (%)	100	100	100

Questiomycin A (**1**) was added into modified SWM-3 medium contained in 6-well polystylene plates or glass vials adjusted to the final concentration of 3, 30, and 300 ng/mL, then kept for 7 days at room temperature. Concentration of compound **1** in 7 days old solution was measured by LC-MS with MRM method. Recovery rate from polystylene plate (%) was calculated from the results of glass vials (100% recovery).
